# Alkaline-Enhanced Poly(Acrylic Acid)/Sodium Alginate/PEO Hydrogels: Structural Modifications and Functional Properties for Agriculture

**DOI:** 10.3390/gels12050395

**Published:** 2026-05-02

**Authors:** Elena Manaila, Gabriela Craciun, Maria Mihaela Manea, Marius Dumitru

**Affiliations:** 1 Electron Accelerators Laboratory, National Institute for Laser, Plasma and Radiation Physics, 077125 Măgurele, Romania; gabriela.craciun@inflpr.ro (G.C.); marius.dumitru@inflpr.ro (M.D.); 2 Multipurpose Irradiation Facility Center—IRASM, Horia Hulubei National Institute for R&D in Physics and Nuclear Engineering, 077125 Măgurele, Romania; mmanea@nipne.ro

**Keywords:** hydrogels, electron beam irradiation, alkaline treatment, water retention, Flory-Rehner analysis, hydrogel network restructuring

## Abstract

This study examines the impact of alkaline treatment on hydrogels composed of acrylic acid (AAc), sodium alginate (SA), and poly(ethylene oxide) (PEO), produced via 5.5 MeV electron beam irradiation, emphasizing swelling behavior and functional performance. Hydrogels were treated with NaOH (0.25 M and 0.50 M) to modulate biodegradability, water retention capacity, and water retention ratio. The materials were characterized in terms of structural, morphological, thermal, and physicochemical properties using FTIR, SEM, and TGA/DSC, along with evaluations of gel fraction, cross-linking density, mesh size, porosity, swelling kinetics, and water retention. FTIR confirmed carboxyl group ionization and polymer chain reorganization, while SEM revealed structural changes, rougher surfaces, and larger pores that facilitate water uptake. Thermal stability of the hydrogels increased, with the T-onset rising from 236 °C in the untreated samples to 451 °C after alkaline treatment. Treatment with 0.25 M NaOH enhanced mesh size (127.97 ± 4.05 nm), porosity (99.74 ± 0.05%), and swelling capacity (428 ± 14 g/g), whereas 0.50 M induced partial degradation and reduced swelling. Despite a significant increase in degradability (>39.49 ± 1.94% after 28 days), treated hydrogels maintained functional performance, showing accelerated water uptake and improved rainwater retention. Overall, alkaline treatment enables tunable structural and functional modifications, optimizing hydrogel performance for agricultural water management.

## 1. Introduction

Due to their cross-linked structure and hydrophilic functional groups, hydrogels form three-dimensional polymer networks capable of absorbing and retaining large amounts of water. In agriculture, they enhance soil water management by absorbing rain- and irrigation-derived water and releasing it slowly to plants, thereby reducing water stress, increasing water use efficiency [1,2], decreasing irrigation intervals, and supporting plant growth under water stress conditions. They are particularly useful in substrates with limited water-holding capacity, such as pots, greenhouses, or seasonal crops, where they promote more uniform water availability. In modern agriculture, hydrogels must balance network stability with biodegradability [3], maintaining water retention during the crop cycle while gradually degrading to prevent the persistence of synthetic residues in soil environments.

Based on their source of origin, hydrogels can be classified as synthetic, such as those based on sodium polyacrylate or polyacrylamide [4], and natural, of biopolymer origin as alginate, chitosan, or modified starch [1,5,6]. In the case of polyacrylate- or polyacrylamide-based hydrogels, partial hydrolysis alters the polymer network structure [7,8,9], leading to increased swelling and water retention, particularly during the initial stages. This behavior is advantageous during critical phases of germination and rapid plant growth and can also promote biodegradation processes. Natural hydrogels are biodegradable [5] and offer the additional benefit of integrating into the soil without accumulating synthetic residues. Moreover, through partial degradation, these hydrogels can release oligomers or fragments [10], which contribute to the stimulation of soil microbiota. Such effects can enhance stress tolerance in plants and delay seedling wilting occurrence up to over a period of approximately ten days [1], positively influencing plant health and root system development.

Sodium alginate has been extensively investigated as a biopolymer for agricultural hydrogel applications due to its water-attracting nature, biocompatible, and biodegradable nature. Previous studies have explored a wide range of alginate-based hydrogel systems with varying compositions and synthesis approaches. For instance, bead-shaped hydrogels based on sodium alginate and poly(acrylic acid), prepared using calcium chloride serving as a crosslinker, were evaluated with and without anionic phosphate and carbonate ions, exhibiting a maximum water absorption of approximately 75% in distilled water [11]. Further developments include graft copolymers of sodium alginate with acrylic and methacrylic acids, obtained via gamma-induced copolymerization in the presence of N,N′-methylenebisacrylamide (5–30 kGy), which achieved water absorption values of up to 300% under optimized conditions (15 kGy, 0.03% cross-linker) [12]. Similarly, superabsorbent hydrogels developed from sodium alginate, gelatin, and acrylamide have been synthesized by gamma irradiation (10–40 kGy), showing gel fractions between 76.8 and 90.3% and equilibrium swelling capacities ranging from 40 to 75.5 g/g, depending on irradiation dose [13]. Notably, post-synthesis modifications such as controlled alkaline hydrolysis (10% KOH at 90 °C) significantly enhanced swelling performance, increasing water uptake from 77.5 g/g to values exceeding 1000 g/g over successive cycles, followed by a decrease due to structural degradation [13]. These studies collectively demonstrate that the swelling behavior of alginate-based hydrogels is strongly influenced by synthesis parameters, cross-linking strategy, and post-treatment processes, which govern network restructuring and water retention capacity. Beyond their swelling performance, sodium alginate hydrogels have also been shown to be biocompatible, biodegradable, and non-toxic to plants and soil [14]. Their functional carboxyl groups contribute to improved soil properties by regulating micronutrient availability [15], influencing organic carbon content [16], and stimulating microbial activity [17]. Additionally, alginate-based microspheres prepared via CaCl_2_-induced gelation exhibit complete biodegradation within approximately 28 days [18], further supporting their suitability for sustainable agricultural applications.

Poly(acrylic acid) is a synthetic polymer well known for its functional carboxyl groups [19]; however, hydrogels composed solely of acrylic acid exhibit low mechanical strength despite high water absorption capacity. To enhance mechanical properties, poly(acrylic acid) is often copolymerized and cross-linked with other polymers [19]. Additionally, poly(acrylic acid) is non-toxic and non-irritant, and its oligomers degrade by up to 80% within 35 days. In a cross-linked polymeric material, up to 4% of the composition may consist of linear poly(acrylic acid), which, although its impact on water retention is limited, promotes biodegradation [20,21].

Poly(ethylene oxide) (PEO) is water-compatible, environmentally degradable, and non-toxic, miscible with sodium alginate for hydrogel preparation. During the synthesis process, hydrogen bonding may occur between the hydroxyl groups of sodium alginate and the ether oxygen atoms of PEO [22], leading to enhanced network stability and improved physical, and mechanical properties as well as water retention capacity [23].

While hydrogels have been widely investigated for agricultural applications, the effects of alkaline treatment on electron beam-irradiated poly(acrylic acid)/sodium alginate/PEO hydrogels remain unexplored, particularly regarding structural modifications, swelling behavior, and water retention in soil.

The purpose of this study was twofold: first, to investigate the impact of alkaline treatment with NaOH solutions (0.25 M and 0.50 M) on the functional properties of AAc/SA/PEO-based hydrogels, synthesized by 5.5 MeV electron beam irradiation; and second, to investigate their potential for sustainable agricultural applications by assessing their degradation behavior and water retention capacity. Electron beam irradiation offers several advantages, including precise process control, the possibility of combining hydrogel formation and sterilization in a single technological step, the use of low concentrations of initiators, crosslinking agents, or other additives, the absence of chemical waste, and improved structural homogeneity of the polymer network [19]. The two concentrations were chosen to highlight the effect of doubling the molarity of the alkaline solution on the performance of the hydrogels. Alkaline treatment alters the hydrogel network structure, leading to measurable changes in swelling, water retention, and functional properties relevant for agriculture.

The polymer network structure was characterized by determining the gel fraction, cross-linking degree, network mesh size, and porosity, while the swelling behavior was assessed through measurements of swelling degree, kinetics, and dynamics. Degradation and water retention properties were evaluated using tests conducted directly in soil. Functional groups and chemical modifications induced by alkaline treatment were analyzed using Fourier-transform infrared (FTIR) spectroscopy. Scanning electron microscopy (SEM) was used to examine the surface morphology, while thermal stability and degradation temperature were investigated using thermogravimetric analysis (TGA) and differential scanning calorimetry (DSC). The swelling behavior in soil was also investigated by evaluating the water holding capacity (WH) and the water retention ratio (WR) over two consecutive cycles in real aqueous environments (rainwater and tap water), rather than conventionally used distilled water. This approach introduces a more application-relevant evaluation framework, offering a realistic assessment of hydrogel performance under agricultural conditions and revealing the influence of water composition on WH and WR.

## 2. Results and Discussion

### 2.1. Structural, Chemical, and Network Characterization of Hydrogels

#### 2.1.1. Structural Characterization by Fourier-Transform Infrared FTIR) Spectroscopy

**Figure 1:**
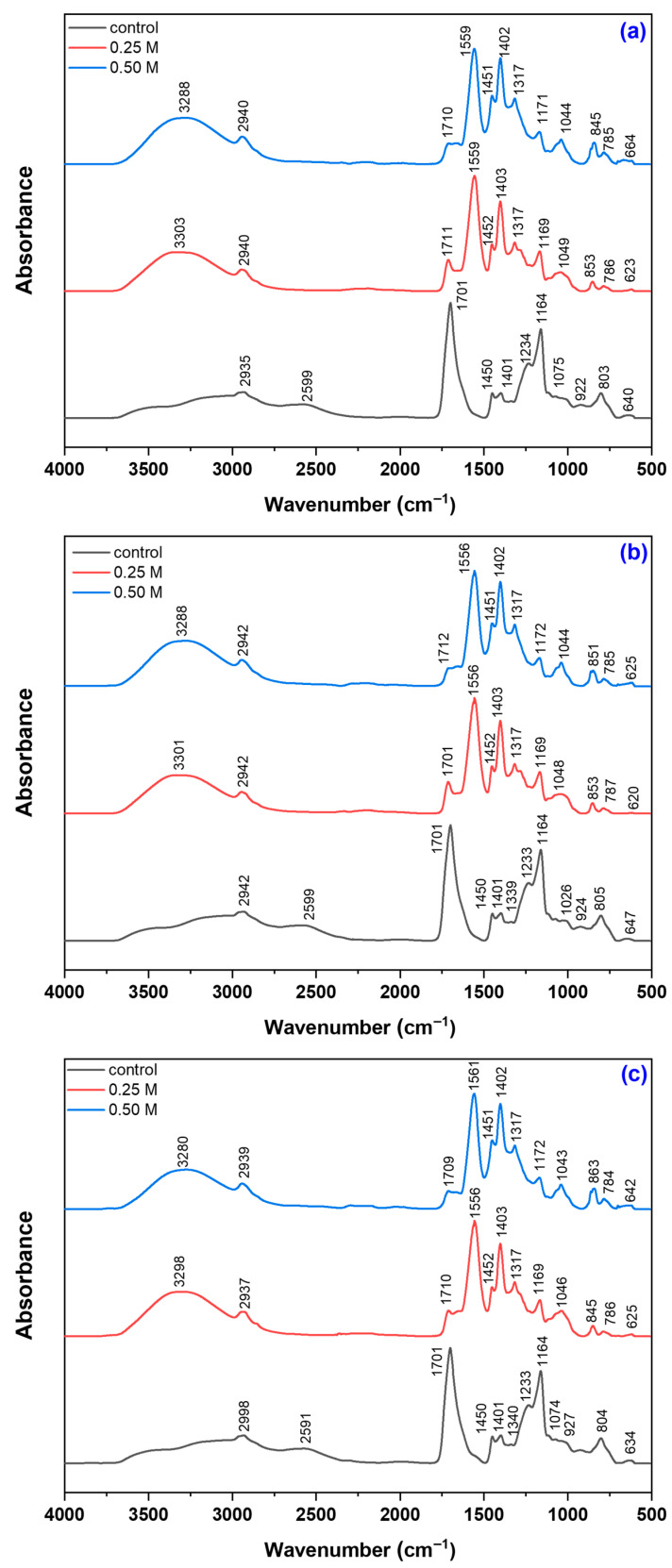
FTIR spectra of hydrogels containing 0.1% potassium persulfate (PPS) obtained at irradiation doses of (**a**) 10 kGy, (**b**) 15 kGy, and (**c**) 20 kGy, before and after NaOH treatment.

To evaluate the effect of alkaline treatment on the structure of hydrogels, FTIR analysis was carried out before and after exposure to NaOH, highlighting modifications of functional groups and network restructuring (Figure 1).

The FTIR spectra shows a broad band in the 3303–3280 cm^−1^ region, attributed to ν(O–H) stretching vibrations, evidencing the presence of hydroxyl groups and hydrogen bonding [24]. This band (~3550–3600 cm^−1^) is broad and has low intensity in untreated samples, whereas in samples treated with alkaline solution, the intensity increases with NaOH concentration, and the band shifts to lower wavenumbers (~3300 cm^−1^), indicating stronger hydrogen bonding and a reorganization of the gel network. The band in the 3000–2940 cm^−1^ region is mainly assigned to ν(C–H) stretching of aliphatic groups (–CH_2_–, –CH–). After alkaline treatment, this band shifts to lower wavelengths (~2940 cm^−1^) and increases in intensity, reflecting changes in the chemical environment of the polymer chains following deprotonation of carboxyl groups and reorganization of intermolecular interactions. This increase also reflects a change in the orientation of carboxylate groups, influencing the hydrogels hydrophilic character [25].

The broad band in the 2598–2591 cm^−1^ region corresponds to ν(O–H) vibrations of the carboxylic –COOH groups involved in hydrogen bonding and combined vibrations [26]. The disappearance of this band after NaOH treatment confirms the deprotonation of COOH groups and the formation of carboxylate COO^−^ groups, also indicating a reorganization of the hydrogel network. The very intense band observed in the 1700–1650 cm^−1^ region is attributed to the ν(C=O) stretching vibration of the carbonyl group in protonated carboxylic acid (–COOH) from poly(acrylic acid), also associated with the stretching of the carboxylate anion (–COO^−^), as evidenced by the sharp peak at ~1701 cm^−1^ and the shoulders at 1662–1656 cm^−1^ [27]. Following alkaline treatment, the band shifts to higher wavenumbers (~1710–1712 cm^−1^) with a strong intensity decrease (from ~0.9998 to 0.2222–0.2706 for 0.25 M NaOH and 0.1573–0.1813 for 0.50 M NaOH), reflecting altered hydrogen bonding and carbonyl microenvironment.

The bands in the 1550–1401 cm^−1^ region are assigned to the asymmetric and symmetric stretching vibrations of carboxylate groups, ν_as_(–COO^−^) and ν_s_(–COO^−^) [28,29]. Following alkaline treatment, the appearance of intense bands at 1563–1556 cm^−1^ (ν_as_(–COO^−^)) confirms deprotonation of carboxylic groups and formation of sodium carboxylate groups within the hydrogel network. The symmetric stretching bands at 1450–1400 cm^−1^ shift slightly (~1452 and ~1403 cm^−1^) and increase markedly in intensity, particularly at higher NaOH concentration (0.4059–0.4257 and 0.7778–0.8003 for 0.25 M, and 0.5458–0.5996 and 0.9041–0.9186 for 0.50 M NaOH, respectively). These spectral changes indicate network reorganization and increased COO^−^ content, consistent with enhanced hydrophilicity and swelling capacity.

The band at ~1340 cm^−1^ (1339–1338 cm^−1^) is attributed to δ(CH_2_) bending vibrations of methylene groups from poly(acrylic acid), PEO, and the guluronic (G) and mannuronic (M) units of sodium alginate, while the band at ~1230 cm^−1^ (1233–1232 cm^−1^) corresponds to ν(C–O–C) glycosidic stretching [25,30]. After alkaline treatment, the ~1340 cm^−1^ band shifts to lower wavenumbers (~1317 cm^−1^) and increases in intensity, indicating structural reorganization of the polymer network, altered M/G interactions, and enhanced ionic interactions due to carboxylate (–COO^−^) formation, leading to greater exposure of C–H groups. In contrast, the decreased intensity of the ~1230 cm^−1^ band suggests partial hydrolysis or modification of glycosidic linkages induced by the alkaline environment. The absorption bands at 1165 cm^−1^, 1074 cm^−1^, and 1025 cm^−1^ are attributed to the asymmetric and symmetric stretching vibrations of the glycosidic bond ν_as_(C–O–C) and ν_s_(C–O–C), as well as to the skeletal vibration ν(C–O), all characteristic of polysaccharides [25].

Alkaline treatment induced pronounced FTIR changes, indicating structural and chemical rearrangements within the polymer network. The band at ~1165 cm^−1^ (C–O–C glycosidic vibrations) shifted slightly to ~1170 cm^−1^ and decreased in intensity, suggesting altered interactions between guluronic (G) and mannuronic (M) units due to carboxyl group ionization and formation of –COO^−^-based ionic interactions. The band at ~1074 cm^−1^ shifted to ~1050 cm^−1^, showing a NaOH-dependent trend (decrease at 0.25 M and increase at 0.50 M), together with similar variations at ~1025 cm^−1^, indicating modifications of ether linkages and saccharide interactions, likely involving partial hydrolysis and/or network reorganization. Overall, changes in C–O–C band intensity reflect a balance between bond cleavage and formation of new ionic interactions, leading to either partial network disruption or enhanced structural stabilization depending on NaOH concentration.

In untreated samples, a band at ~930 cm^−1^ (ν(C–C) and ν(C–O)) is observed [28]. After alkaline treatment, it decreases and is replaced by a band at 860–840 cm^−1^, attributed to ρ(CH_2_) of PEO [31], indicating a transition to more ordered (helical) chain conformations. This reflects a conformational reorganization of the polymer network driven by carboxyl group ionization, which increases electrostatic repulsion and promotes chain extension and ordering of PEO segments.

In the 800–700 cm^−1^ region, assigned to anomeric C–H deformations of “β-D-mannuronic”(~819 cm^−1^) and “α-L-guluronic” (~782 cm^−1^) residues [32,33,34,35], the untreated sample shows a dominant band at ~803 cm^−1^, while alkaline-treated samples exhibit new bands at ~853, ~785, and ~700 cm^−1^. Although overall intensities decrease relative to the untreated state, they increase with NaOH concentration, with stronger signals at 0.50 M than at 0.25 M, indicating concentration-dependent structural reorganization and altered contributions from M/G units. Alkaline treatment induces cleavage of glycosidic bonds via a β-elimination mechanism [36,37], leading to depolymerization of the chains and the formation of unsaturated end units, altering the chemical structure of the anomeric bonds and decreasing the content of mannuronate and guluronate units [37]. Additionally, washing the hydrogels with distilled water after alkaline treatment removes a portion of the low molecular weight fractions generated during alkaline degradation, explaining the decrease in intensity of these absorption bands.

The band in the 647–633 cm^−1^ region corresponds to collective skeletal vibrations of the polymer network [30], mainly involving ν(C–O) and ν(C–O–C) modes. This skeletal region reflects backbone vibrations rather than specific functional groups. The band is present in the untreated hydrogel, indicating the intrinsic backbone structure of PEO and sodium alginate. After alkaline treatment, its intensity increases, attributed to carboxyl group ionization and subsequent network reorganization, leading to a more ordered polymer conformation.

#### 2.1.2. Morphological Analysis by Scanning Electron Microscopy (SEM)

**Figure 2:**
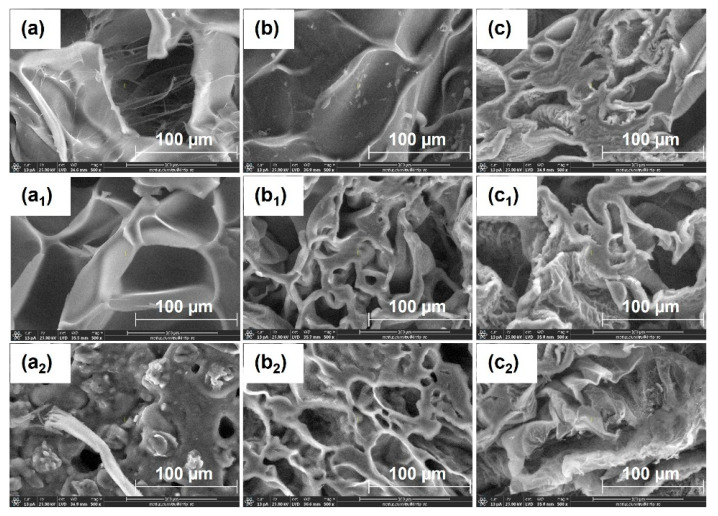
SEM micrographs (100× magnification) of freeze-dried hydrogels synthesized at 10 kGy with varying PPS concentrations: (**a**–**c**) untreated hydrogels containing 0.1, 0.2 and 0.3% PPS; (**a_1_**–**c_1_**) corresponding hydrogels treated with 0.25 M NaOH; and (**a_2_**–**c_2_**) corresponding hydrogels treated with 0.50 M NaOH.

SEM micrographs (Figure 2) clearly illustrate the structural changes induced by NaOH treatment on hydrogels. As shown in Figure 2, the untreated hydrogels exhibit a relatively uniform morphology, characterized by a porous network with interconnected macropores and thin walls, suggesting a lamellar (“slice-like”) structure [38,39,40]. The pore size distribution indicates a relatively homogeneous structure. Additionally, a gradual reduction in pore size is observed with increasing PPS concentration, suggesting an increase in cross-linking density. After alkaline treatment, the hydrogels exhibit significant morphological changes, characterized by a more heterogeneous structure with larger cavities and macropores, indicating network reorganization [40]. These changes are attributed to the alkaline hydrolysis of carboxylic groups (–COOH) in acrylic acid units, leading to the formation of carboxylate groups (–COO^−^). This process increases the negative charge density within the network, enhancing electrostatic repulsion and promoting hydrogel expansion.

In addition, the partial disruption of physical interactions between alginate and PEO chains contributes to network relaxation and the formation of larger pores. Furthermore, the hydrogel surface becomes rougher, and the resulting microstructures provide additional active sites for interaction with water molecules, thereby enhancing both water uptake and retention [13].

#### 2.1.3. Thermal Analysis (TGA/DSC)

**Figure 3:**
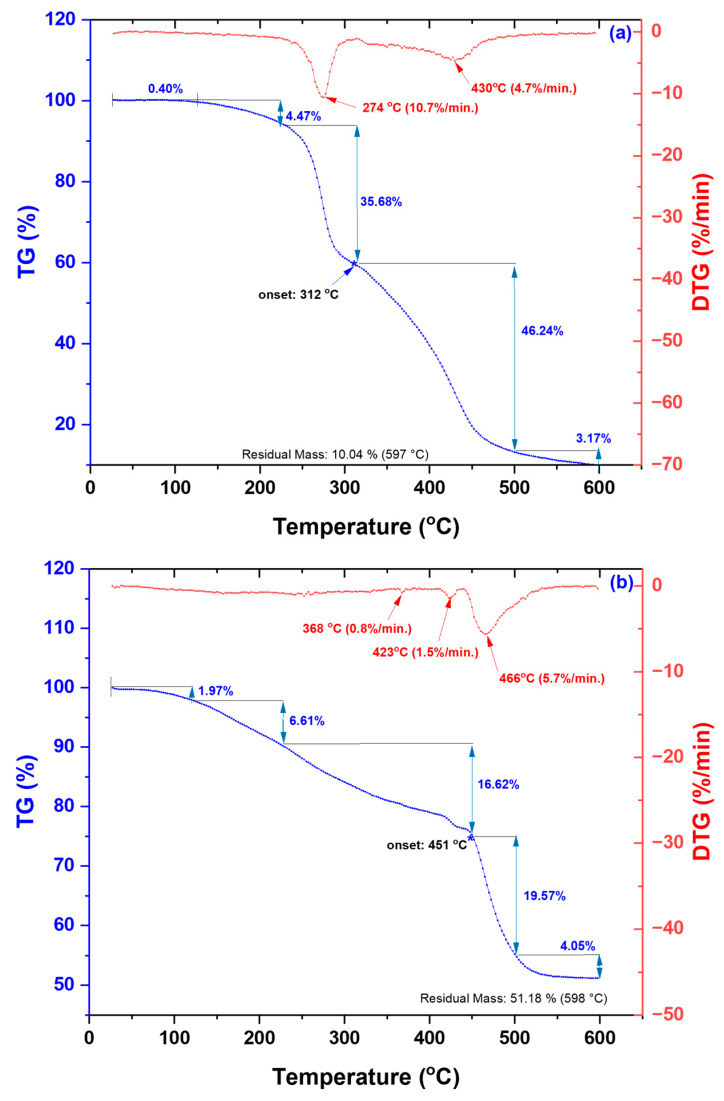

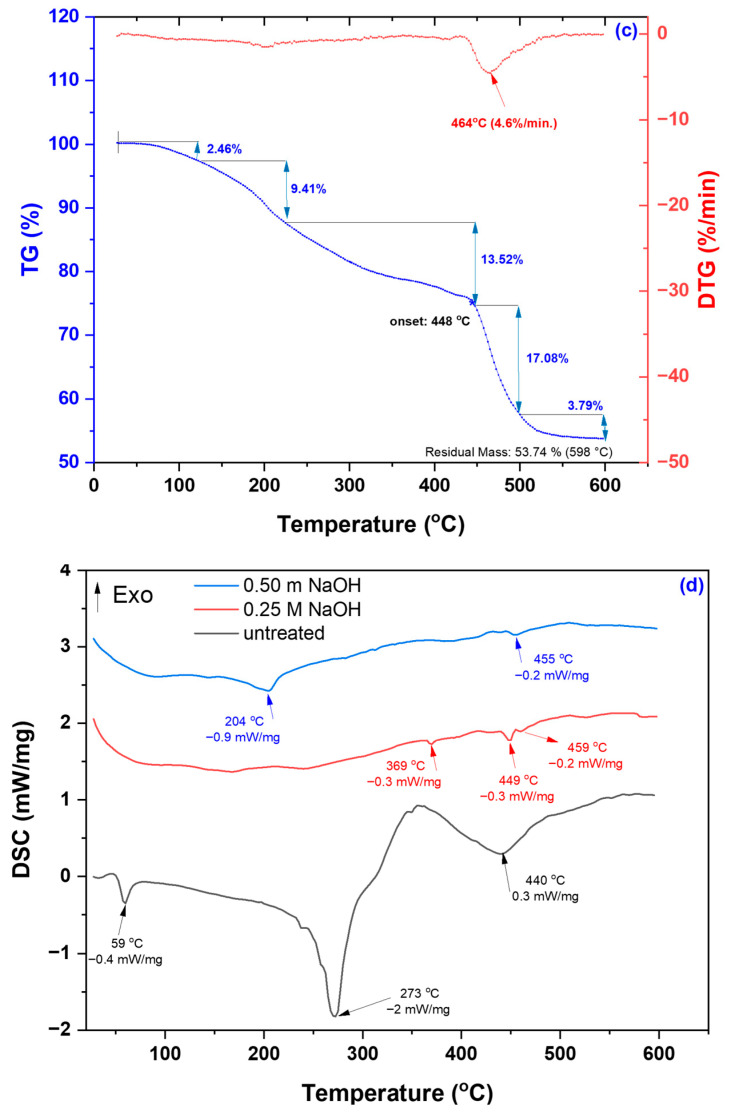
TG, DTG, and DSC curves of the hydrogel obtained at 10 kGy with 0.1% PPS: (**a**) TG–DTG curves for the untreated sample; (**b**) TG–DTG curves for the sample treated with 0.25 M NaOH; (**c**) TG–DTG curves for the sample treated with 0.50 M NaOH; (**d**) DSC curves for all samples.

Thermal characterization was performed by thermogravimetric analysis (TGA) under a nitrogen atmosphere. The TG thermograms and their corresponding DTG curves are presented in Figure 3a–c, while the DSC curves are shown in Figure 3d.

The untreated hydrogel exhibits a typical multi-step degradation profile characteristic of polymeric networks containing both physically and chemically bound water. As shown in Figure 3a, an initial minor mass loss (~0.4%) below 120 °C is attributed to the evaporation of physically adsorbed water [27,41], consistent with the weak endothermic DSC peak observed at ~59 °C. The 120–250 °C range, associated with a mass loss of ~4.5%, is related to the removal of strongly bound water and the onset of degradation of thermally sensitive functional groups. These processes include incipient decarboxylation of acrylic acid units, partial dehydration of pyranose rings in alginate, and partial cleavage of C–O–C bonds [42,43,44,45], accompanied by the release of low-molecular-weight compounds without significant backbone degradation. In the 250–350 °C interval, extensive degradation of the polymer network occurs, involving pronounced decarboxylation, decomposition of pyranose rings, and chain scission via C–O bond cleavage [46,47]. The main degradation stage begins at a T-onset of ~312 °C and is associated with a significant mass loss (~35.7%), corresponding to polymer backbone decomposition. This stage is supported by a DTG peak at ~274 °C (−10.7%/min), indicating the maximum degradation rate. The 350–500 °C range corresponds to advanced degradation and carbonization processes, involving decomposition of poly(acrylic acid) chains, degradation of sodium alginate, and formation of carbonaceous residues, including sodium carbonate [46,47]. For the untreated sample, a thermal event is observed at ~430 °C in this region. The DSC signal confirms complex degradation processes involving bond scission and structural rearrangements. The relatively low residual mass (~10%) indicates limited char formation and confirms the predominantly organic and thermally labile nature of the hydrogel matrix.

The hydrogel treated with 0.25 M NaOH exhibits markedly different thermal behavior compared to the untreated sample, indicating alkaline-induced structural reorganization. The initial mass loss (~2.0%) below 120 °C is higher than that of the untreated hydrogel, reflecting increased water retention due to enhanced hydrophilicity and network swelling. The degradation process extends over a broader temperature range with overlapping stages, suggesting structural heterogeneity within the polymer network. The main degradation region (~300–400 °C) becomes less pronounced, indicating partial stabilization of thermally labile components. DTG peaks at ~368 °C and ~423 °C reveal distinct degradation pathways, likely associated with modified polymer chains and newly formed cross-linked structures. A shift in the main degradation stage to higher temperatures is observed, with T-onset at ~451 °C, confirming enhanced thermal stability after alkaline treatment. This behavior is attributed to alkali-induced structural rearrangements, including chain reorganization, increased intermolecular interactions, and the formation of thermally more stable domains. The residual mass (~51.2%) is significantly higher than in the untreated hydrogel, indicating enhanced char formation and improved thermal stability. Overall, NaOH treatment promotes network densification and stabilization, resulting in increased resistance to thermal degradation.

The hydrogel treated with 0.5 M NaOH exhibits further changes in thermal behavior compared to both the untreated and 0.25 M-treated samples. The initial mass loss (~2.5%) below 120 °C confirms increased water uptake, consistent with enhanced hydrophilicity induced by stronger alkaline treatment. The degradation remains multi-step; however, the intermediate stage (~200–300 °C) becomes more pronounced, as evidenced by a DSC peak at ~204 °C, suggesting the formation of newly generated or partially degraded domains during alkaline treatment. The main degradation stage occurs at high temperatures, with T-onset at ~448 °C and a DTG maximum at ~464 °C, confirming improved thermal stability relative to the untreated hydrogel. However, the slightly lower T-onset compared to the 0.25 M sample suggests that excessive alkalinity may induce partial chain scission, leading to coexistence of stabilized and weakened network regions. The residual mass (~53.7%) is the highest among all samples, indicating enhanced char formation and the development of highly thermally stable structures. Overall, higher NaOH concentrations promote concurrent cross-linking and condensation processes, resulting in a denser and more thermally resistant hydrogel network.

In the temperature range of 500–590 °C, for both untreated and treated hydrogels, the final carbonization of the hydrogels occurs, characterized by the removal of residual volatile fragments and stabilization of the carbonized phase [42]. Low mass losses are recorded (2.28% for the untreated sample and 4.06% and 3.79% for the samples treated with 0.25 M NaOH and 0.50 M NaOH, respectively), indicating that most of the organic degradation processes take place at lower temperatures. The remaining mass consists predominantly of inorganic residues and carbonized fraction.

A comparative analysis of the three samples highlights the significant effect of alkaline treatment on the structural organization and thermal stability of the hydrogels. The final residual mass reflects the overall thermal stability of the system. The untreated hydrogel exhibits a typical degradation pattern with a low residual mass (~10%), consistent with hydrophilic polymer networks reported in the literature [44,48,49]. In contrast, the treated samples show substantially higher residual masses of ~51.2% and ~53.7% for 0.25 M and 0.50 M NaOH, respectively. This increase confirms enhanced stability of the hydrogel network after alkaline treatment, indicating that the conversion of carboxylic groups into carboxylate ions contributes to improved thermal resistance and higher char retention at elevated temperatures [50,51]. In addition, the onset degradation temperatures (T-onset) clearly demonstrate the effect of alkalization on thermal stability. The untreated sample exhibits a T-onset of ~312 °C, indicating the onset of thermal degradation at relatively low temperature. In contrast, NaOH-treated samples show significantly improved stability, with T-onset shifting to ~451 °C and ~448 °C for 0.25 M and 0.50 M NaOH, respectively. This behavior is consistent with previous reports describing alkaline-induced structural rearrangements, increased cross-linking density, and the formation of more thermally stable domains in polymeric and polysaccharide-based hydrogels [52]. A comparison between the 0.25 M and 0.5 M NaOH-treated samples reveals a dual effect of alkaline treatment. While moderate treatment (0.25 M) leads to maximum thermal stabilization, a further increase in NaOH concentration (0.5 M) results in a slight decrease in the onset temperature. This suggests a balance between cross-linking and degradation mechanisms, where excessive alkaline conditions may promote partial chain scission, as reported in previous studies [53].

This significant increase in thermal stability can be attributed to the combined structural modifications induced by alkaline treatment. Specifically, NaOH promotes the deprotonation of acrylic acid groups, facilitating the formation of ionic interactions with Na^+^ ions that act as additional crosslinking points. This ionization process leads to a more interconnected polymer network and is supported by the FTIR spectra (Figure 1). Furthermore, alkaline treatment enhances intermolecular interactions within the hydrogel matrix, including hydrogen bonding and chain entanglement between sodium alginate, PEO, and ionized acrylic acid. In addition, the presence of Na^+^ ions induce electrostatic screening effects, which facilitate polymer chain rearrangement and contribute to the formation of a more compact network structure. As a result of these combined effects, the crosslinking density increases while polymer chain mobility is significantly reduced, thereby delaying the onset of thermal degradation. Although the increase observed in this study is higher than typically reported values [54,55,56], the overall trend remains consistent with literature findings, where enhanced intermolecular interactions and network densification lead to improved thermal stability and higher degradation temperatures.

The DSC analysis (Figure 3d) corroborates the thermal behavior observed in the TG/DTG curves. The untreated sample exhibits endothermic transitions at 56, 174, 251, 298, and 430 °C, corresponding to water loss, decarboxylation, and stepwise degradation of the polymer chains [44,45]. The sample treated with 0.25 M NaOH shows shifted endothermic transitions at 369, 449, and 459 °C, indicating a thermally more stable network due to the neutralization of carboxylic groups. The sample treated with 0.50 M NaOH exhibits only two endothermic transitions at 204 and 455 °C, demonstrating that major degradation is concentrated at high temperatures, while preliminary losses are minimal. These observations indicate that alkalization enhances the thermal stability of the hydrogels and modifies the degradation mechanism, reducing intermediate stages and favoring simultaneous degradation at elevated temperatures [46,47].

### 2.2. Gel Fraction, Network Parameters, and Swelling of Hydrogels

**Figure 4:**
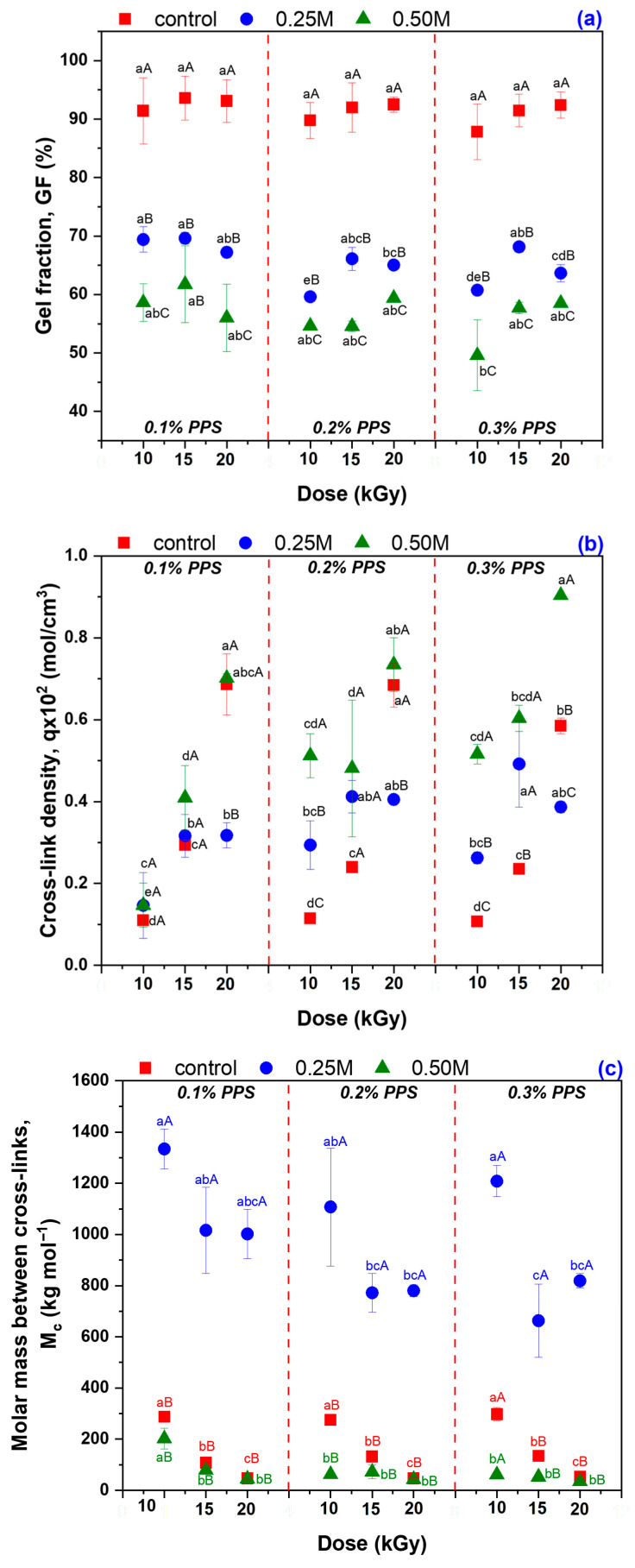

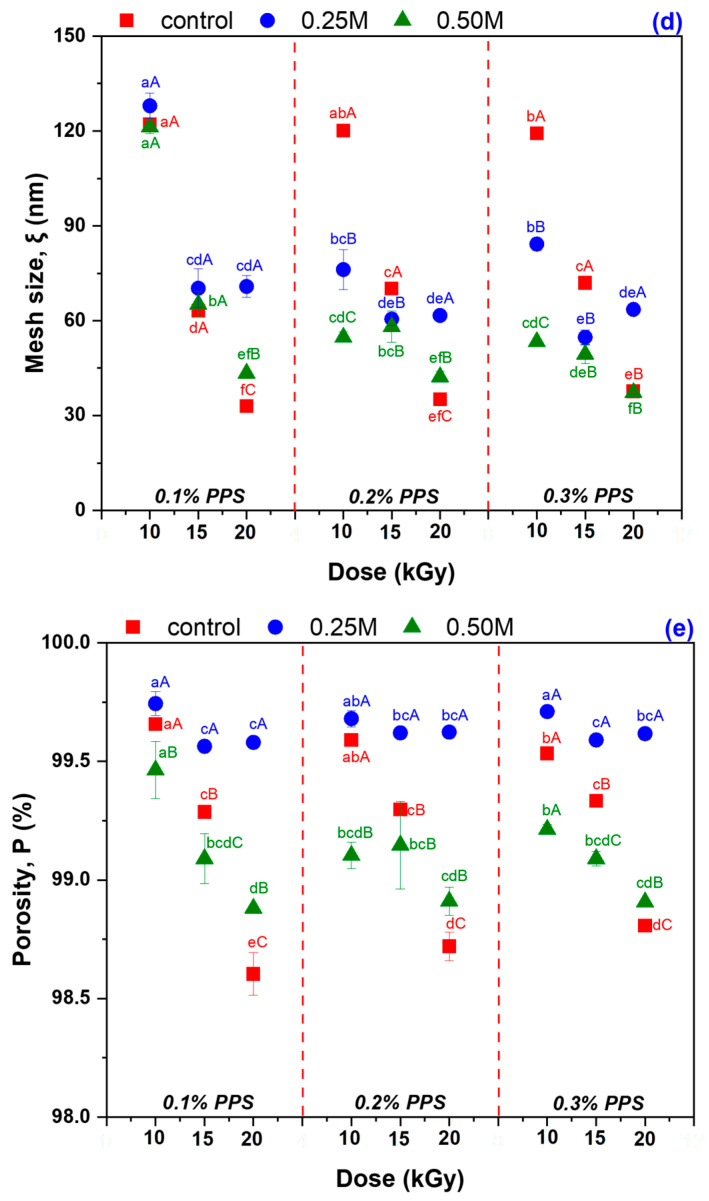
Physicochemical properties of the hydrogels, (**a**) gel fraction, GF, (**b**) cross-linking density, q, (**c**) average molar mass between cross-links, $M_{c}$, (**d**) mesh size, ξ, and (**e**) porosity, P. Data points labeled with different superscript letters (lowercase and uppercase) indicate statistically significant differences (*p* ≤ 0.05).

The investigation of the hydrogels’ physicochemical properties, reflecting their structure, behavior, and functionality, is presented in Figure 4. The reported values represent the mean of three independent measurements. Data points labeled with different superscript letters (lowercase and uppercase) indicate statistically significant differences (*p* ≤ 0.05), as determined by one-way ANOVA followed by Tukey’s post hoc test. Specifically, different lowercase superscripts denote significant differences within each formulation set, defined by PPS concentration and irradiation dose (0.1% PPS: 10, 15, and 20 kGy; 0.2% and 0.3% PPS: 10–20 kGy) across the three experimental conditions: untreated, 0.25 M NaOH-treated, and 0.50 M NaOH-treated hydrogels. In contrast, uppercase superscripts indicate significant differences among the three treatment conditions for each hydrogel formulation.

Hydrogels obtained by irradiation typically exhibit high gel fractions (>75%), reflecting the formation of a stable three-dimensional network through radical cross-linking reactions [57]. The results presented in Figure 4a indicate that, for the hydrogels analyzed in this study, the gel fraction exceeds 87% for all three PPS concentrations.

Electron beam irradiation promotes cross-linking via a radical mechanism, forming a predominantly covalent three-dimensional network. However, due to the balance between cross-linking and chain scission, the resulting network contains regions of moderate density and potential structural heterogeneities. Moreover, the gel fraction slightly increases with increasing irradiation dose, suggesting a higher density of stable cross-linking nodes. This confirms the efficiency of irradiation-generated radicals in promoting covalent bond formation between polymer chains. NaOH treatment induces significant, concentration-dependent modifications of the polymer network. The gel fraction decreases from 87.82 to 93.59% to 59.62–69.62% (0.25 M NaOH) and 49.59–61.75% (0.50 M NaOH), indicating partial polymer chain degradation in the alkaline medium. This structural loosening is reflected in increased mesh size and porosity, particularly for the 0.25 M-treated samples.

NaOH treatment induces complete deprotonation of acrylic acid carboxylic groups (–COOH, converted into –COO^−^), increasing the negative charge density within the network [58]. This generates strong electrostatic repulsion between polymer chains and increases internal osmotic pressure [59], thereby enhancing the swelling degree. In weakly cross-linked networks, the resulting internal stresses may lead to rupture of weaker covalent bonds. In addition, irradiation may introduce oxidized groups or residual peroxide structures on PEO and alginate chains, which are susceptible to alkaline degradation [60,61]. Sodium hydroxide further promotes chain scission, reducing the molecular weight of polymer segments and converting part of the cross-linked fraction into soluble species. The cross-linking degree varies from 0.107 to 0.686 × 10^−2^ mol/cm^3^ to 0.146–0.492 × 10^−2^ mol/cm^3^ (0.25 M NaOH) and 0.147–0.904 × 10^−2^ mol/cm^3^ (0.50 M NaOH), indicating concurrent stabilization of certain network regions via secondary cross-linking and degradation of others at higher alkalinity. The decrease in gel fraction observed after alkaline treatment, despite the increase in cross-linking density, can be rationalized in terms of structural reorganization of the polymer network. Under alkaline conditions, hydrolysis and deprotonation of functional groups occur, leading to significant changes in the network charge density and chain interactions. FTIR analysis provided evidence supporting the deprotonation of functional groups, confirming the chemical modification of the hydrogel structure. The experimental results presented in Figure 4b show a decrease in gel fraction following alkaline treatment of the hydrogels, while the cross-linking density estimated via the Flory–Rehner model exhibits an apparent increase. At first glance, these observations may seem contradictory; however, they reflect different aspects of the hydrogel network structure. The gel fraction represents the proportion of insoluble material and is influenced by the degradation of weakly cross-linked segments, whereas the cross-linking density calculated from the Flory-Rehner model depends on the polymer volume fraction in the swollen gel and the swelling degree of the remaining insoluble network [62]. After alkaline treatment and the removal of soluble segments, the remaining network becomes more compact and denser, explaining the apparent increase in cross-linking density. Thus, despite the reduction in total gel mass, the ratio of cross-linking nodes to the effective volume of the remaining gel increases, indicating a selective restructuring of the hydrogel network: weakly cross-linked segments are removed, while stable and densely cross-linked nodes remain dominant.

Alkaline treatment significantly affected the average molar mass between cross-links, $M_{c}$, as shown in Figure 4c. The initial hydrogels exhibited $M_{c}$ values of 46.37–297.45 kg/mol. After treatment with 0.25 M NaOH, $M_{c}$ increased markedly to 663.01–1334.37 kg/mol, indicating a reduction in cross-linking density, likely due to partial hydrolysis of network bonds and carboxyl group ionization, which induces electrostatic repulsion and network expansion. In contrast, treatment with 0.50 M NaOH reduced $M_{c}$ to 35.01–201.90 kg/mol, suggesting an apparent increase in cross-linking density or network compaction driven by alkaline degradation and structural reorganization. A higher cross-linking density corresponds to a more compact network with reduced chain mobility. Accordingly, the average distance between cross-linking points (mesh size, *ξ*) decreases with increasing cross-link density, as shown in Figure 4d, due to reduced spatial separation between junctions [63]. For NaOH-treated hydrogels, the mesh size increases from 32.93 to 122.15 nm to 54.77–127.97 nm at 0.25 M, indicating network expansion at moderate alkalinity, whereas it decreases to 37.27–121.30 nm at 0.50 M, reflecting partial network collapse at higher concentration.

The porosity of the hydrogels, initially 98.60–99.66%, increases to 99.56–99.74% at 0.25 M NaOH and decreases to 98.88–99.46% at 0.50 M NaOH, as shown in Figure 4e, indicating that structural changes modulate the free volume available for water retention. Both porosity and mesh size are highly sensitive to NaOH concentration. At 0.25 M, their increase is attributed to carboxyl group ionization and enhanced electrostatic repulsion between polymer chains, promoting network expansion and the formation of wider diffusion channels [64,65]. These changes enhance water retention and facilitate transport within the hydrogel. In contrast, at 0.50 M NaOH, the decrease in porosity and mesh size suggests partial polymer degradation and network collapse under strongly alkaline conditions, as previously reported for pH-responsive hydrogels [64,66]. Under these conditions, the network free volume decreases, leading to reduced water-holding capacity and impaired diffusion properties.

All these results confirm the pH-dependent behavior of hydrogels based on polymers with ionizable groups. The structural changes induced by NaOH demonstrate that the hydrogel responds differently depending on the solution concentration: moderate concentrations expand the network, increasing mesh size and porosity, whereas higher concentrations weaken the network, induce polymer chain degradation, and increase structural variability, thereby affecting the hydrogel properties [65,67].

**Figure 5:**
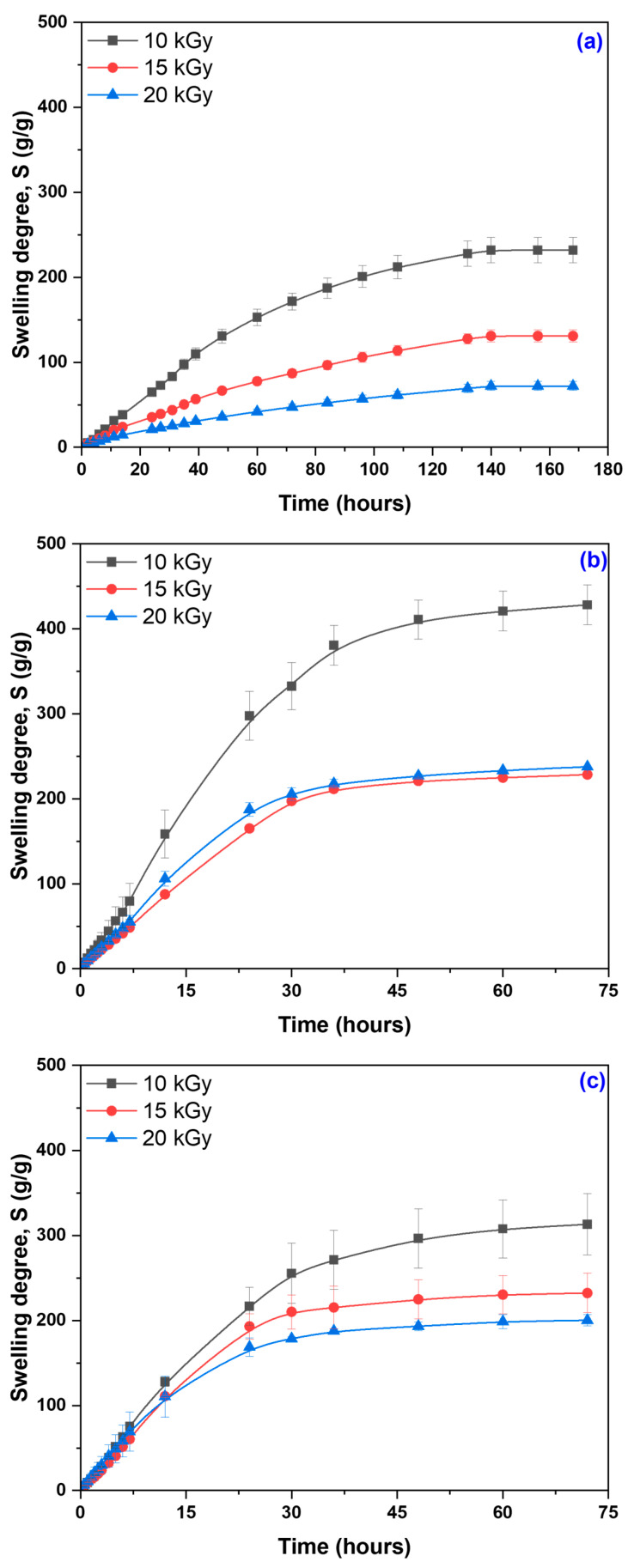
Swelling of hydrogels prepared with 0.1% PPS: (**a**) untreated samples; (**b**) samples treated with 0.25 M NaOH; (**c**) samples treated with 0.50 M NaOH.

**Figure 6:**
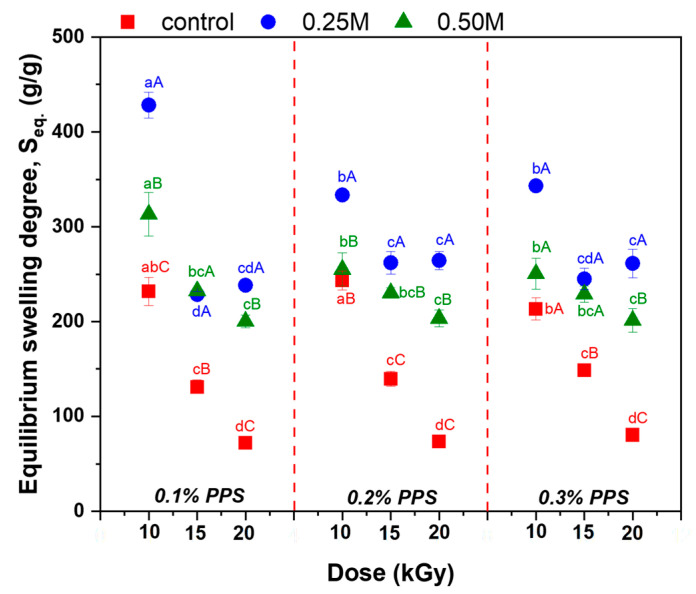
Swelling at equilibrium, $S_{eq}$. Data points labeled with different superscript letters (lowercase and uppercase) indicate statistically significant differences (*p* ≤ 0.05).

The results of the immersion experiments in distilled water for the hydrogel containing 0.1% PPS, together with the equilibrium swelling values (*S_eq._*) for all hydrogel variants, are presented in Figure 5 and Figure 6. The corresponding data for hydrogels containing 0.2% and 0.3% PPS are provided in the Supplementary Material (Figures S1 and S2).

**Figure 7:**
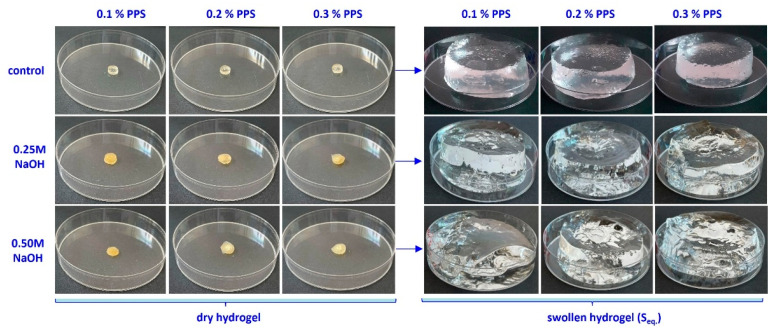
Images of hydrogels obtained at 10 kGy, untreated and treated with alkaline solution, after the swelling process.

**Figure 8:**
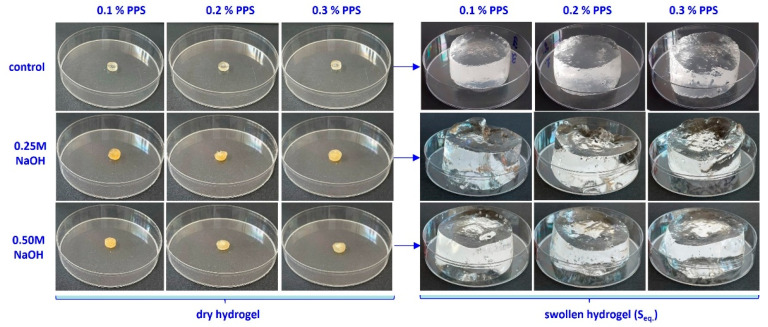
Images of hydrogels obtained at 15 kGy, untreated and treated with alkaline solution, after the swelling process.

**Figure 9:**
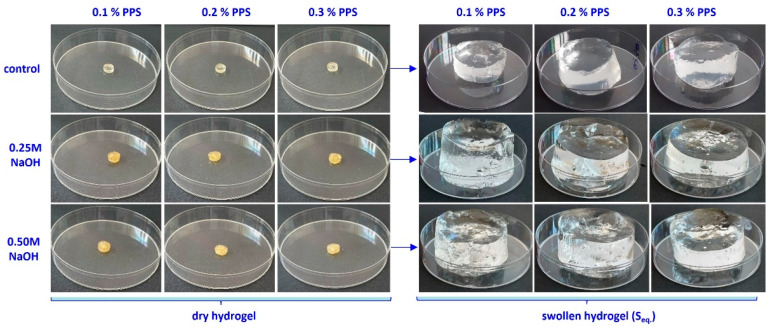
Images of hydrogels obtained at 20 kGy, untreated and treated with alkaline solution, after the swelling process.

The swelling degree is strongly influenced by cross-linking density and is inversely proportional to it [68]. In turn, cross-linking density depends on both irradiation dose and PPS concentration. The highest water absorption values were obtained at 10 kGy. Increasing PPS concentration resulted in comparable swelling degrees across all irradiation doses. Although cross-linking density increases with PPS concentration, the mesh size decreases, likely due to competing degradation effects arising from the cascade of free radicals generated during irradiation [69]. Generation of a large number of active free-radical sites on the SA chain facilitates the grafting of acrylic acid onto the biopolymer backbone. The slight increase in the swelling degree with PPS concentration can be attributed to a self-crosslinking effect arising from bimolecular termination reactions [70,71]. Images of the hydrogels in dry state and after equilibrium swelling are shown in Figure 7, Figure 8 and Figure 9.

The structural changes induced by NaOH treatment are reflected in the equilibrium swelling behavior of the hydrogels. The initial hydrogels exhibit swelling capacities of 72–243 g/g, which increase to 229–428 g/g and 200–313 g/g after treatment with 0.25 M NaOH and 0.50 M NaOH, respectively. The higher swelling observed at 0.25 M indicates a more pronounced expansion of the polymer network under moderate alkaline conditions. The increase in swelling is directly correlated with mesh size enlargement, as additional water uptake separates macromolecular chains and increases the length of elastic segments between cross-linking points. Enhanced porosity further increases the free volume within the three-dimensional network. In contrast, at 0.50 M NaOH, the reduced swelling suggests limited network expansion due to partial chain degradation and/or structural reorganization of the cross-linked network. This is accompanied by decreased mesh size and tighter chain packing, leading to lower porosity. Consequently, structural compaction negatively affects both water retention capacity and transport properties. Overall, the swelling behavior exhibits a clear dependence on NaOH concentration. Moderate NaOH levels promote network expansion, leading to increased mesh size, enhanced porosity, and higher swelling capacity. In contrast, excessive NaOH concentrations induce polymer chain degradation, partial collapse of the network structure, and increased structural heterogeneity, ultimately impairing the hydrogel’s swelling performance. These observations are consistent with literature reports on polysaccharide-based hydrogel systems [11,12,13,14,15,16,17,18]. In particular, sodium alginate and its derivatives have been shown to display a broad range of swelling capacities, from tens to over 1000 g/g, depending on polymer composition, cross-linking strategy, and the extent of chemical modification, highlighting the critical role of structural and chemical parameters in governing water uptake.

### 2.3. Swelling Kinetics: Analysis Using Kinetic Models and Diffusion Behavior

Swelling, a key property of hydrogels, is influenced by factors such as polymer composition and structure, cross-linking method and degree, pore size, and the number of hydrophilic functional groups. To evaluate hydrogel swelling behavior, kinetic models based on diffusion and polymer relaxation mechanisms [15,72,73] have been widely applied. The corresponding results obtained in this study are summarized in Table 1, Table 2, Table 3 and Table 4.

Modeling the experimental data using first- and second-order kinetic models, together with diffusion analysis, allows identification of the dominant transport mechanisms in hydrogels before and after alkaline modification. Alkaline treatment significantly alters the kinetic and diffusion parameters, reflecting pronounced structural changes in the polymer network. The first-order swelling rate constant ($k_{1,s}$) describes the rate of water uptake when swelling follows first-order kinetics, where the rate is proportional to the difference between equilibrium and time-dependent swelling [74], indicating a predominantly diffusion-controlled process. For untreated samples, $k_{1,s}$ values are relatively low, but increase markedly after alkaline treatment, with higher values observed at increased NaOH concentrations. The higher values at 0.50 M suggest that increased deprotonation of carboxylic groups enhances electrostatic repulsion, leading to network expansion and increased permeability, which facilitates water transport. In contrast, the second-order swelling rate constant ($k_{2,s}$) shows a more pronounced increase at 0.25 M NaOH compared to 0.50 M, indicating an optimal ionization range at moderate alkalinity. This model reflects coupled diffusion–polymer relaxation processes [74]. Higher $k_{2,s}$ values correspond to rapid initial swelling followed by a decrease in the swelling rate, whereas lower values indicate slower and more uniform swelling due to restricted chain mobility. At moderate alkalinity, the network achieves a balance between ionization and structural integrity, favoring efficient relaxation and transport. At higher NaOH concentration, electrostatic screening and possible structural degradation reduce the contribution of second-order swelling behavior.

The diffusion exponent (n) and kinetic constant (k) were determined using the Korsmeyer–Peppas model. The diffusion mechanism was interpreted based on n, which describes the mode of water transport within the hydrogel. In general, n = 0.45 corresponds to Fickian diffusion, where swelling is governed by solvent diffusion with negligible polymer relaxation; 0.45 < n < 0.89 indicates anomalous (non-Fickian) transport governed by both diffusion and polymer relaxation; n = 0.89 corresponds to Case II transport dominated by polymer relaxation; and n > 0.89 describes Super Case II transport, typically associated with highly extensible networks or osmotic effects [75,76]. For untreated hydrogels, the diffusion exponent n indicates anomalous transport and Super Case II, with comparable contributions from diffusion and polymer relaxation. After NaOH treatment, n shifts toward values characteristic of Case II-like behavior, suggesting a transition to a mechanism dominated by structural relaxation of the polymer network. In parallel, the kinetic constant k increases significantly after alkaline treatment, indicating accelerated water uptake. This behavior becomes more pronounced at higher NaOH concentration. Overall, after alkaline treatment, the swelling mechanism shifts toward Case II-like behavior, where polymer relaxation plays a dominant role. Enhanced hydrophilicity facilitates solvent penetration, while network relaxation governs overall transport, resulting in a coupled diffusion–relaxation mechanism with reduced diffusional resistance [77]. The same conclusions are supported by the diffusion coefficient (*D*). Alkaline treatment leads to a substantial increase in D, indicating a more expanded polymer network driven by deprotonation, electrostatic repulsion, and osmotic effects [77,78].

The correlation between SEM morphology, FTIR analysis, swelling behavior, and kinetic modeling provides a comprehensive understanding of the effect of alkaline treatment on hydrogel performance. SEM images reveal an increase in mesh size and a more open porous network after alkaline treatment, which facilitates water diffusion into the polymer matrix. FTIR analysis confirms significant chemical changes in the carboxylic groups, where the decrease in the C=O stretching band of COOH (~1715–1725 cm^−1^) and the appearance of COO^−^ asymmetric (~1550–1580 cm^−1^) and symmetric (~1400–1450 cm^−1^) stretching vibrations indicate deprotonation and formation of carboxylate groups. In addition, the increase in the O–H stretching band (~3550–3600 cm^−1^) further supports enhanced hydrogen bonding interactions with water molecules. These structural and chemical modifications result in improved water penetration and higher swelling capacity. Consistently, swelling kinetics show increased rate constants and diffusion coefficients after alkaline treatment, indicating faster water uptake. Overall, the combined SEM–FTIR–swelling–kinetics results demonstrate a clear structure–chemistry–property relationship governing hydrogel behavior.

### 2.4. Hydrogel Degradation

The ability of hydrogels to undergo degradation under aerobic and anaerobic conditions, mediated by microbial enzymatic activity, represents a key property of these materials. Biodegradation involves the breakdown of organic substances and is closely related to material characteristics such as water solubility and absorption capacity [79].

**Figure 10:**
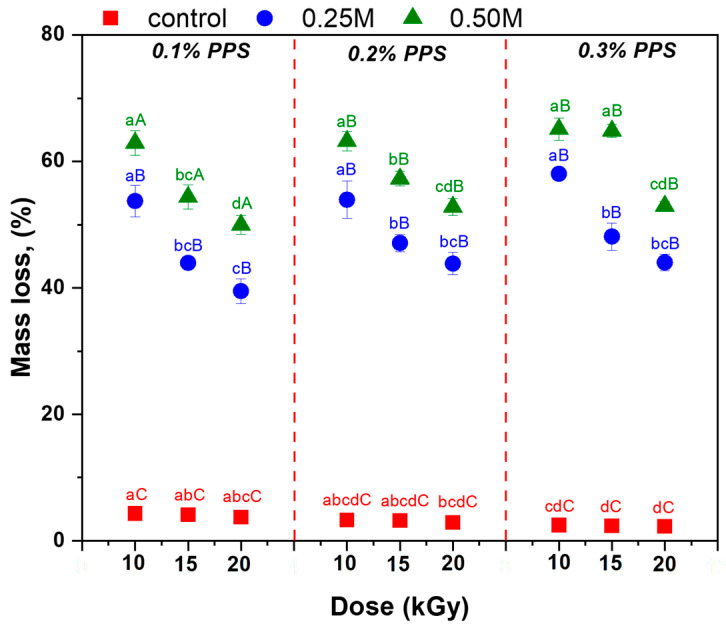
Mass loss (%) of untreated and NaOH-treated hydrogels after 28 days of soil burial. Data points labeled with different superscript letters (lowercase and uppercase) indicate statistically significant differences (*p* ≤ 0.05).

**Figure 11:**
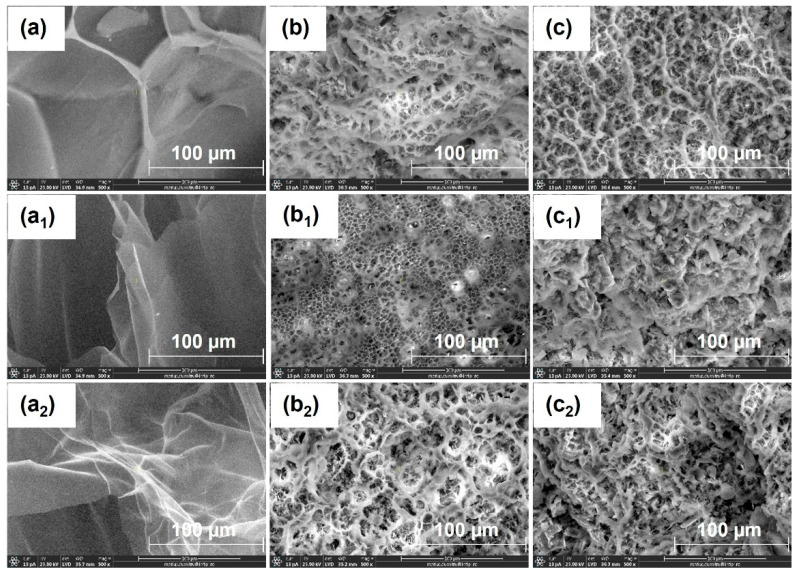
SEM micrographs (100× magnification) of freeze-dried hydrogels obtained at 10 kGy with different PPS concentrations after degradation: (**a**–**c**) initial hydrogels containing 0.1, 0.2 and 0.3% PPS; (**a_1_**–**c_1_**) corresponding samples treated with 0.25 M NaOH; (**a_2_**–**c_2_**) corresponding samples treated with 0.50 M NaOH.

The biodegradability of both untreated and NaOH-modified hydrogels was evaluated using the soil burial method. Each sample was weighed and buried for 28 days, and the degree of degradation was determined based on mass loss (Figure 10). To investigate the effects of degradation on the internal structure, the morphology of the lyophilized hydrogels was examined using Scanning Electron Microscopy (SEM) after the biodegradation period, and the corresponding images are presented in Figure 11.

Analysis of mass loss after 28 days of soil burial highlights the significant effect of alkaline treatment on the stability of hydrogels cross-linked by irradiation. Untreated hydrogels exhibit minimal degradation (2.26–4.32%), indicating a relatively stable network. In contrast, NaOH treatment markedly increases degradability, with mass losses of 39.43–58.04% for 0.25 M and 49.96–65.11% for 0.50 M NaOH. This behavior is attributed to weakening of intra-network interactions and deprotonation of carboxylic groups, resulting in a more expanded structure susceptible to solubilization and chemical cleavage under alkaline conditions. These results demonstrate that NaOH concentration is a key parameter for tuning the balance between structural stability and degradability. Although partial structural integrity is retained, the release of more mobile or weakly bound components may affect mechanical stability and swelling behavior, while generating biodegradable species potentially beneficial for plant growth.

Sodium alginate is biodegradable and non-toxic, undergoing complete degradation within 28 days [18], while its carboxylic groups contribute to improved soil properties, micronutrient regulation, organic carbon balance, and stimulation of microbial activity [11,15,16,17]. Similarly, poly(acrylic acid) is non-toxic and partially biodegradable, with oligomers degrading up to 80% within 35 days; residual linear fractions also contribute to overall degradation [20]. The observed degradation levels are consistent with those reported for similar biopolymer- and poly(acrylic acid)-based hydrogels [80,81,82,83]. SEM analysis (Figure 11) reveals significant morphological changes after soil burial compared to the initial structure (Figure 2), confirming progressive network degradation. Changes in pore size and morphology reflect this process: untreated hydrogels exhibit enlarged interchain spaces, whereas NaOH-treated samples display finer pores and filamentous structures within cavities [11]. These observations indicate that alkaline treatment modifies network architecture and influences the degradation behavior of hydrogels in soil.

### 2.5. Hydrogel Water-Holding Capacity and Water Retention Ratio

**Figure 12:**
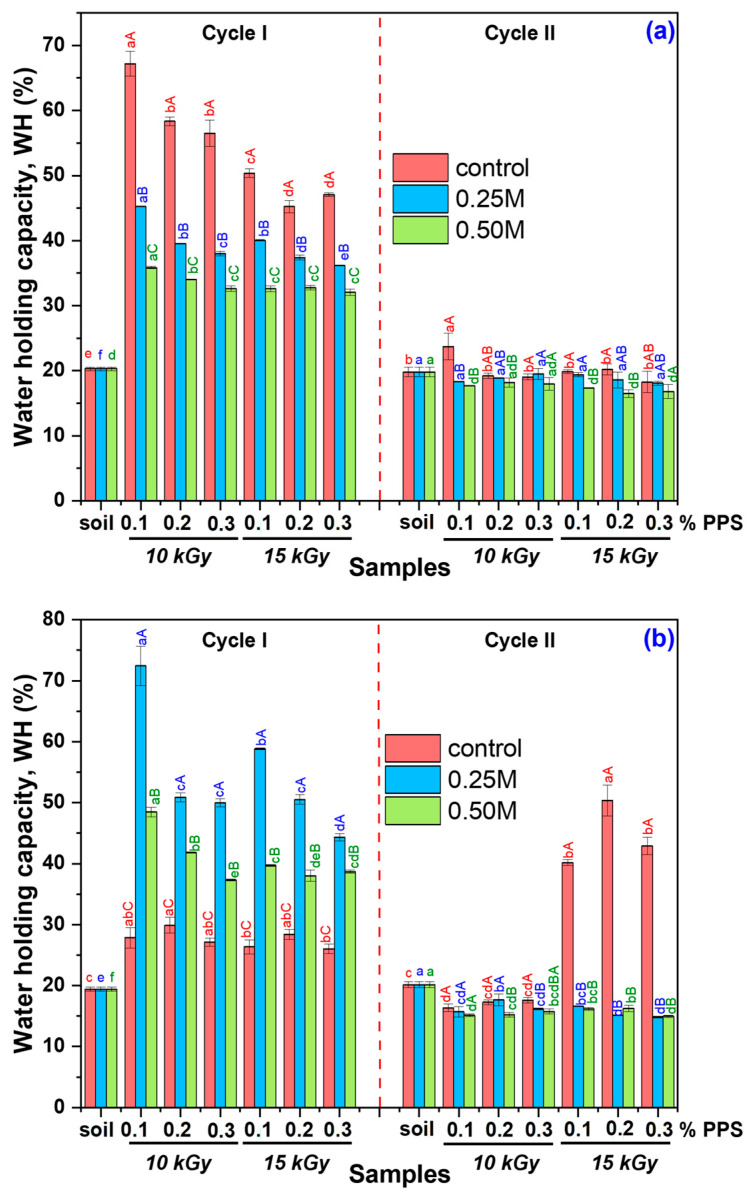
Water-holding capacity (WH) of hydrogels in (**a**) tap water and (**b**) rainwater. Data points labeled with different superscript letters (lowercase and uppercase) indicate statistically significant differences (*p* ≤ 0.05).

The swelling behavior of the synthesized hydrogels after alkaline treatment in soil was evaluated in terms of water-holding capacity (WH) and water retention ratio (WR) over two consecutive cycles, using tap water and rainwater as swelling media. The corresponding WH results are presented in Figure 12a,b.

Data points marked with different superscript letters indicate statistically significant differences (*p* ≤ 0.05), as determined by one-way ANOVA followed by Tukey’s post hoc test. Lowercase letters denote differences within each PPS concentration and irradiation dose (0.1–0.3% PPS, 10 and 15 kGy) under the three conditions (untreated, 0.25 M NaOH, and 0.50 M NaOH), while uppercase letters indicate differences between treatment conditions for each hydrogel type. Although alkali-treated hydrogels exhibit higher water-holding capacity in distilled water (Figure 6), lower WH values are observed in tap water during the first cycle (Figure 12a) compared to the control samples. This behavior is attributed to the presence of dissolved ions, which increase the ionic strength of the swelling medium, leading to charge screening of functional groups and a reduction in osmotic pressure driving swelling. Furthermore, divalent cations (e.g., Ca^2+^ and Mg^2+^) can form additional ionic interactions with anionic groups in the polymer network, promoting partial network contraction and consequently reducing water-holding capacity [84,85].

A behavior similar to that observed in distilled water was also found in rainwater during the first cycle (Figure 12b), where the lower concentration of dissolved ions reduces charge screening effects, allowing electrostatic repulsion between ionized groups to be maintained and resulting in higher water-holding capacity (WH) [86]. In the second cycle, however, untreated samples exhibit higher WH values than NaOH-treated hydrogels. This behavior can be attributed to structural adaptation: during the first cycle, initially more rigid polymer chains in untreated samples undergo rearrangement and relaxation, leading to increased porosity and improved water uptake in the subsequent cycle. In contrast, NaOH-treated hydrogels undergo significant expansion during the first cycle and reach a relatively stable structure, limiting further increases in WH. The decrease in WH observed in cycle II for treated hydrogels, in both tap water and rainwater, can be associated with partial network degradation, which reduces water retention capacity. This effect likely arises from a combination of factors, including mechanical constraints imposed by the soil matrix, pore occupation by degradation products, and ionic interactions that restrict swelling. Additionally, hydrogel–soil interactions and changes in pore architecture may alter pore size distribution, affecting water flow and retention in the soil [87,88].

**Figure 13:**
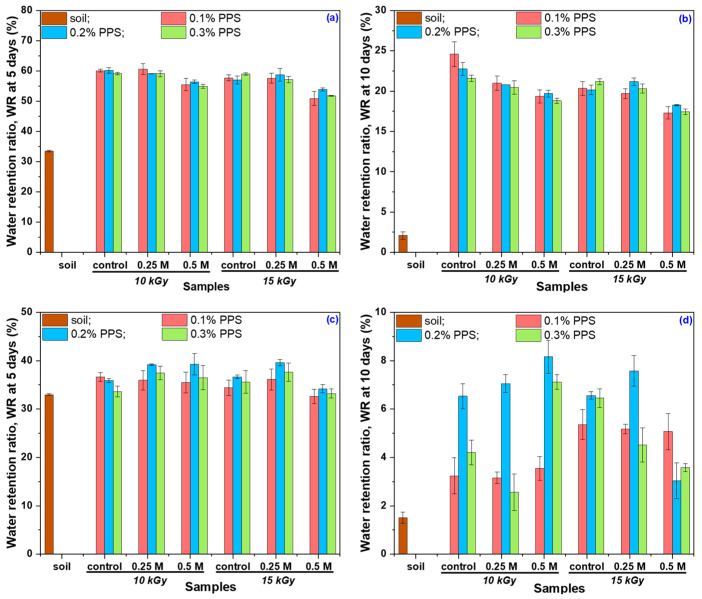
Water retention ratio (WR) of the hydrogels in tap water measured during Cycle I: (**a**) 5 days, (**b**) 10 days; and during Cycle II: (**c**) 5 days, (**d**) 10 days.

**Figure 14:**
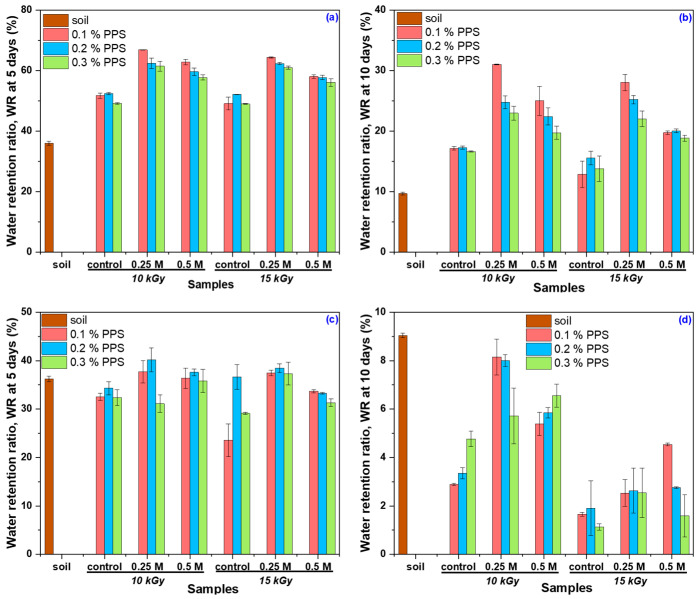
Water retention ratio (WR) of the hydrogels in rainwater measured during Cycle I: (**a**) 5 days, (**b**) 10 days; and during Cycle II: (**c**) 5 days, (**d**) 10 days.

In the subsequent stage of the experiment, the water retention ratio (WR) was monitored over a period of 10 days in both testing cycles, using tap water and rainwater as swelling media. The corresponding WR results for the tested period are provided in the Supplementary Material (Figures S3 and S4). The water retention ratio (WR) of the hydrogels in tap water and rainwater, measured over two testing cycles and reported after 5 and 10 days, is presented in Figure 13 and Figure 14.

The results (Figure 13) show that, during the first testing cycle, untreated hydrogels exhibited higher WR values in tap water compared to alkali-treated samples. This phenomenon is attributed to dissolved ions in tap water, which interact with functional groups in the polymer network, leading to charge screening and disruption of the internal osmotic balance. As a result, the hydrogel structure becomes less stable, promoting network contraction and enhanced degradation, consistent with both reduced WR values and the higher degradation degree (Figure 10). In the second cycle, WR values were generally comparable among samples, although some notable differences were observed. Alkali-treated hydrogels showed moderate to substantial increases in WR relative to the control, with the magnitude of improvement depending on both NaOH concentration and initiator (PPS) content. Overall, these findings indicate that alkaline treatment alters the hydrogel network structure and, consequently, its water retention capacity.

To evaluate the influence of water type on retention, the WR in rainwater (Figure 14) reflects the combined effects of alkaline treatment, irradiation dose, and PPS concentration on hydrogel behavior. In all cases, alkali-treated hydrogels exhibited higher WR values than untreated samples.

During Cycle I, hydrogels irradiated at both doses showed noticeable improvements in WR following alkaline treatment, with more pronounced increases observed for samples treated with the lower NaOH concentration, particularly at longer immersion times. A similar trend was maintained across irradiation conditions, indicating a consistent response to alkaline modification. In Cycle II, WR values remained higher for alkali-treated samples compared to the control, although the magnitude of improvement varied depending on treatment conditions. The most significant enhancements were generally observed after prolonged immersion, suggesting a time-dependent stabilization of the hydrogel network. Notably, the highest WR increases were consistently recorded for hydrogels with the lowest PPS content. Overall, these results demonstrate that irradiation dose and initiator concentration significantly influence hydrogel water retention, particularly in low-ion-content media such as rainwater.

Although NaOH-treated hydrogels exhibit significantly higher degradability compared to untreated samples (>40% over 28 days), this does not compromise their functionality during critical stages of plant development, such as seed germination [89]. In the initial application period, these materials show enhanced swelling and higher water retention in rainwater, ensuring rapid water availability for seeds and young plants. While partial degradation may initially appear as a structural limitation, it can provide beneficial effects. Increased porosity and polymer chain mobility facilitate water and oxygen diffusion in soil, improving aeration and moisture retention [90]. Moreover, oligomeric degradation products from poly(acrylic acid) and alginate can be metabolized by soil microorganisms, thereby stimulating root development [89,91]. This dual behavior imparts multifunctional properties, combining water storage capacity with plant biostimulatory effects. Analysis over two successive swelling–deswelling cycles shows that treated samples reach a stabilized structure after the first cycle, maintaining their water retention performance during the second cycle. In contrast, initially more rigid untreated samples undergo structural rearrangement and may exhibit improved water retention in the second cycle, highlighting the adaptive nature of the hydrogel network.

In conclusion, NaOH treatment provides hydrogels with controlled degradability and multifunctional properties, combining rapid water availability with stimulation of the soil microbiota and root development. These features make them suitable for the initial stages of germination and short-cycle crops [86], demonstrating their potential for modern, sustainable, and innovative agriculture.

## 3. Conclusions

Alkaline treatment with NaOH significantly modulated the physicochemical, structural, thermal, and functional properties of AAc/SA/PEO hydrogels synthesized via 5.5 MeV electron beam irradiation. FTIR analysis revealed extensive chemical changes induced by NaOH, confirming network ionization via disappearance of the –COOH band (~2595 cm^−1^) and appearance of COO^−^ stretching bands (1563–1556 and 1452–1403 cm^−1^), while shifts in ν(C=O), ν(C–O–C), and CH_2_ bands indicated ordered PEO conformations and increased hydrophilicity, especially at 0.50 M NaOH. SEM revealed that untreated hydrogels had uniform pores (35–120 nm), thin walls, and lamellar morphology, while 0.25 M NaOH increased pore size (54–128 nm) and surface roughness, indicating polymer chain relaxation, and 0.50 M induced partial degradation, pore reduction (37–121 nm), and network compaction. TGA/DSC analyses confirmed enhanced hydrogel thermal stability after alkaline treatment, with T-onset increasing from 236 °C to ~448–451 °C and residual mass from 16.85% to 51–54%, alongside reduced degradation steps and merged DTG peaks. Structural characterization showed that 0.25 M NaOH reduced gel fraction (59–70%) but increased mesh size, porosity (99.56–99.74%), and equilibrium swelling (229–428 g/g), while 0.50 M reduced gel fraction (50–62%), mesh size (37–121 nm), porosity (98.88–99.46%), and swelling (200–313 g/g), reflecting partial chain degradation. Despite lower gel fraction, cross-linking density increased, indicating selective retention of highly cross-linked regions. Swelling kinetics and diffusion analyses demonstrated enhanced rate constants, higher diffusion coefficients, and a shift from anomalous to Case II-like transport, indicating diffusion-controlled swelling with reduced structural constraints. Soil burial tests showed minimal degradation of untreated hydrogels (2–4% mass loss), while 0.25 M and 0.50 M treatments increased degradation to 39–58% and 50–65%, respectively, with SEM confirming morphological reorganization. Alkaline treatment enhanced water-holding capacity (WH) and water retention ratio (WR) across two successive soil swelling–deswelling cycles. Hydrogels exhibited superior retention in rainwater due to lower ionic strength, whereas tap water reduced swelling and retention via ionic interactions, demonstrating tunable multifunctionality and adaptability under varying environmental conditions.

In conclusion, alkaline treatment allowed precise tuning of hydrogel structure, swelling behavior, thermal stability, and biodegradability. Moderate alkalization (0.25 M NaOH) optimized network expansion, porosity, and water retention, while higher alkalization (0.50 M) induced partial degradation and compaction, providing a versatile strategy for designing hydrogels with tailored properties for sustainable agricultural applications.

## 4. Materials and Methods

### 4.1. Materials

Acrylic acid, AAc. (Mw = 71.08 g/mol, density = 1.13 g/cm^3^), sodium alginate SA (Mw = 120,000–190,000 g/mol, viscosity = 15–25 cP, 1% in water), potassium persulfate, PPS (Mw = 270.32 g/mol, density = 2.477 g/cm^3^) as a reaction initiator, polyethylene oxide PEO (Mw = 300,000 g/mol, density = 1.210 g/cm^3^), and sodium hydroxide, NaOH (Mw = 39.99 g/mol, density = 2.13 g/cm^3^) were purchased from Merck KGaA, Darmstadt, Germany, and supplied by Redox, Bucharest, Romania, and used without further purification.

### 4.2. Synthesis of Hydrogels by E-Beam Irradiation

The polymeric solutions were prepared following the procedure described in our previous work [92]. Three solutions containing identical concentrations of sodium alginate (0.5%), acrylic acid (20%), and polyethylene oxide (0.1%) were prepared. Sodium alginate (SA) and polyethylene oxide (PEO) were dissolved in distilled water under continuous magnetic stirring (200 rpm), at room temperature (23 ± 2 °C) for 2 h, until a homogeneous solution was obtained. Subsequently, acrylic acid (AAc) was added dropwise under continuous stirring (150 rpm), at room temperature (23 ± 2 °C) for 30 min, yielding a uniform SA/PEO/AAc mixture. The resulting mixture was then divided into three equal portions, to which 0.1%, 0.2%, and 0.3% PPS were added, respectively, to produce the final hydrogel formulations. The polymer solutions were irradiated to form hydrogels at the Electron Accelerator Laboratory using the linear electron accelerator (ALID 7, 5.5 MeV), owned by NILPRP. The irradiation geometry, determined by the diameter of the tube used for hydrogel processing, was set to 10 mm, according to our previous study [93]. Compared with previous experiments, the irradiation dose rate was increased because of its effect on polymer network formation. The solutions were placed in medical syringes with a diameter of 10 mm and irradiated with electron beam doses of 10, 15, and 20 kGy at a dose rate of 1.1–1.2 kGy/min under atmospheric conditions and at room temperature (23 ± 2 °C). Electron beam dosimetry was performed using a graphite calorimeter provided by DTU Health Tech, High Dose Reference Laboratory, Roskilde, Denmark, which serves as the primary standard for electron beam dosimetry. After irradiation, the resulting hydrogels were kept at room temperature for 24 h, then removed from the syringes in cylindrical shape, washed with ethanol and distilled water to remove unreacted chemicals, cut into discs with a height of 3–4 mm, and dried in air for 24 h, followed by drying in a laboratory oven at 55 °C for 24–48 h until a constant weight was reached. The dried hydrogels were then stored in covered containers until further use for characterization and testing.

### 4.3. Functionalization of Hydrogels with Sodium Hydroxide

For functionalization, dried hydrogels were immersed in aqueous solutions of 0.25 M and 0.5 M sodium hydroxide (NaOH) for 24 h. The swollen samples were subsequently washed with distilled water to remove any residual NaOH from the surface. They were then air-dried for 24–48 h, followed by oven-drying at 55 °C for 24–48 h until reaching constant weights. Finally, the samples were stored in covered containers until further use for characterization and testing.

**Figure 15:**
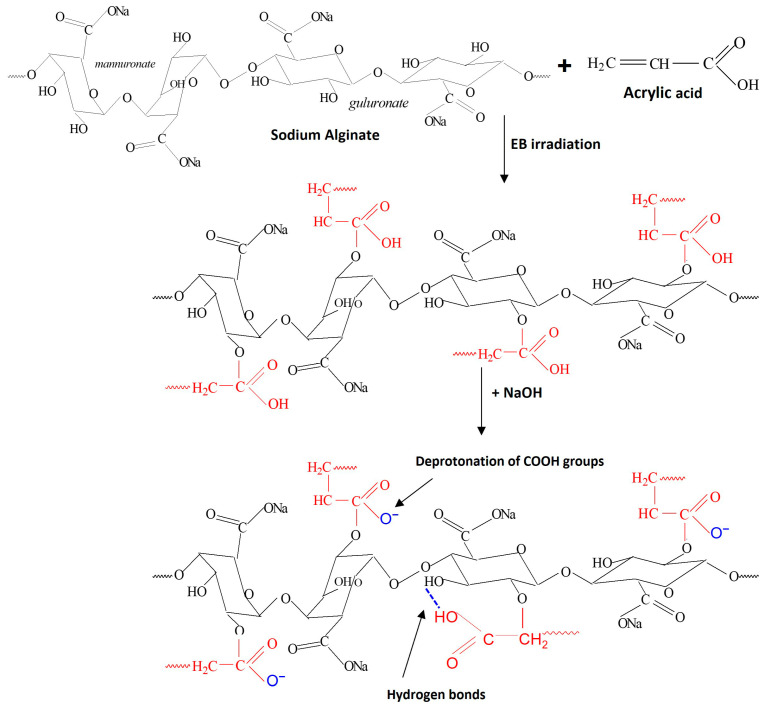
Schematic representation of hydrogel synthesis by irradiation and subsequent NaOH treatment.

The synthesis of the hydrogels by electron beam irradiation and their subsequent modification with NaOH is schematically presented in Figure 15.

### 4.4. Hydrogels Characterization Before and After Sodium Hydroxide Treatment

#### 4.4.1. Sol–Gel Analysis

Dried samples ≈ 0.2 g (*m_i_*) were immersed in distilled water at room temperature (23 ± 2 °C) for 72 h to remove the soluble fraction. After immersion, the samples were then air-dried for 6 days and then dried in a laboratory oven at 55 °C for 48 h until a constant mass was obtained ($m_{f}$). The gel fraction GF (%) was calculated using Equation (1) [94]:

(1) $$GF\;\left(\%\right)=\;\frac{m_{f}}{m_{i}}\times 100,$$

#### 4.4.2. Swelling Analysis

Swelling dynamics and equilibrium swelling degree were determined by immersing the hydrogels in distilled water at room temperature (25 °C). The mass increase relative to the initial mass ($m_{i}$) was recorded at predetermined time intervals ($m_{t}$ at time t) until equilibrium mass was obtained ($m_{eq}$_._). The swelling degree (S, %) and the equilibrium swelling degree ($S_{eq}$, %), were calculated using Equations (2) and (3) [95,96]:

(2) $$S\;\left(\%\right)=\;\frac{m_{t}-m_{i}}{m_{i}}\times 100,$$

(3) $$S_{eq.}\;\left(\%\right)=\frac{m_{eq.}-m_{i}}{m_{i}}\times 100,$$

#### 4.4.3. Network Structure Characterization

Network parameters of the hydrogels, namely the average molecular weight between two cross-links ($M_{c}$), cross-linking density (q), mesh size (ξ), and porosity (P) were evaluated based on swelling data. The cross-link density (q), defined as the mole fraction of cross-linked units, was determined using Equation (4) from the ratio of the average molar mass between cross-links ($M_{c}$), calculated according to the Flory–Rehner theory, to the molar mass of the repeating unit ($M_{r}$) [95,97,98,99]. $M_{c}$ and $M_{r}$ were obtained from Equations (5) and (6). The polymer volume fraction in the swollen gel ($\upsilon_{2,s}$) and the Flory–Huggins polymer–solvent interaction parameter (χ) were determined using Equations (7) and (8) [100,101]:

(4) $$q=\;\frac{M_{c}}{M_{r}},$$

(5) $$M_{c}=-V_{s}d_{p}\frac{\upsilon_{2,s}^{1/3}-\frac{\upsilon_{2,s}}{2}}{\ln\left(1-\upsilon_{2,s}\right)+\upsilon_{2,s}+\chi \upsilon_{2,s}^{2}},$$

(6) $$M_{r}=\frac{\left(m_{SA}\times M_{SA}\right)+\left(m_{AAc}\times M_{AAc}\right)+\left(m_{PEO}\times M_{PEO}\right)}{m_{SA}+m_{AAc}+m_{PEO}},$$

(7) $$\upsilon_{2,s}={\left[1+\frac{d_{p}}{d_{s}}\left(\frac{m_{g}}{m_{i}}-1\right)\right]}^{-1},$$

(8) $$\chi =\frac{\ln\left(1-\upsilon_{2,s}\right)+\upsilon_{2,s}}{\upsilon_{2,s}^{2}},$$

where $V_{s}\;$ is the molar volume of solvent (water) (18 cm^3^ mol^−1^); $d_{p}$ is the polymer density (g cm^−3^); $d_{s}$ is the density of solvent (g cm^−3^); $m_{SA}$, $m_{AAc}$, and $m_{PEO}$ are the masses of sodium alginate, acrylic acid, and poly(ethylene oxide), respectively; and $M_{SA}$, $M_{AAc}$, and $M_{PEO}$ are the corresponding molar masses (g mol^−1^).

The mesh size (ξ) and porosity (P) of the hydrogels were determined according to Equations (9) and (10) [102,103], allowing a quantitative evaluation of the scaffold structural characteristics.

(9) $$\xi =\upsilon_{2,s}^{-1/3}l\;\sqrt{\frac{2C_{n}M_{c}}{M_{r}}},$$

(10) $$P\;\left(\%\right)=\frac{V_{d}}{1-V_{d}}\times 100,$$

where l  is the length of the C–C bond along the polymer backbone (0.154 nm); $C_{n}$ is the Flory characteristic ratio; $V_{d}$ is the volume fraction of water at equilibrium. The value of $C_{n}$ was estimated as the weighted average of the characteristic ratios of poly(acrylic acid), sodium alginate, and poly(ethylene oxide) based on their molar ratios in the hydrogel network: poly(AAc) = 6.7 [102], SA = 21.1 [103], and PEO = 4.98 [104].

#### 4.4.4. Swelling Kinetics

The first-order kinetic model (Equation (11)), after integration under the initial conditions (S = 0 at t = 0, S  = S at time t), is written in the form of Equation (12) [105]:

(11) $$\frac{dS}{dt}=\;k_{1,S}\left(S_{eq.}-S\right),$$

(12) $$lnW=k_{1,S}\;\;where\;W=\frac{S_{eq.}}{S_{eq.}-S},$$

where $k_{1,s}$ is the first-order swelling rate constant, while S and $S_{eq}$ denote the swelling degree at time t and at equilibrium, respectively.

A second-order equation (Equation (13)), based on the equilibrium degree of swelling, can also be applied [95]. After integration with initial conditions (S = 0 at t = 0, S = S at time t), it yields Equation (14).

(13) $$\frac{dS}{dt}=k_{2,S}\;{\left(S_{eq.}-S\right)}^{2},$$

(14) $$\frac{t}{S}=A+Bt,where\;A=r_{0}=\frac{1}{k_{2,S}\times S_{eq.}^{2}}v\;\mathrm{and}\;B=\frac{1}{S_{eq.}},\;$$

where A (or $r_{0}$) denotes the initial swelling rate, $k_{2,s}$ is the second-order swelling rate constant, and B corresponds the reciprocal of the maximum swelling degree ($S_{eq}$) [95].

For both first- and second-order swelling kinetics, ln W versus t and t/S versus t plots were constructed [95]. The slopes and intercepts of these plots were used to determine the first- and second-order swelling rate constants ($k_{1,s}$ and $k_{2,s}$), the initial swelling rate ($r_{0}$), and the theoretical equilibrium swelling ($S_{eq}$) [95].

The diffusion behavior and swelling mechanisms of water n the hydrogels were furter evaluated by applying Equation (15), which is equivalent to Equation (16) for the first 60% of the swelling curves [105,106].

(15) $$S_{swp\;}=\frac{m_{t}-m_{i}}{m_{i}}=kt^{n},$$

(16) $$lnS_{swp}=nlnt+lnk$$

where $S_{swp}$, $m_{t}$, and $m_{i}$ are the swelling ratio at time t, the mass of the swollen hydrogel at time t, and the initial dry hydrogel mass, respectively; k is the swelling constant, and n is the swelling exponent, which reflects the water transport mechanism.

For the hydrogels, ${lnS}_{swp}$ versus lnt (from Equation (16)) was plotted, and the swelling exponent (n) and swelling constant (k) were determined from the slope and intercept of the resulting lines, respectively. The value of n indicates the water transport mechanism, following Fickian kinetics [107]. The swelling–time curves of the hydrogels in water were also used to calculate the diffusion coefficients (D) using Equation (17). This approach is valid only for the first 60% of the swelling process [95].

(17) $$S=4{\left[\frac{D}{\pi xr^{2}}\right]}^{1/2}t^{1/2},$$

where S, D, r and t are the swelling ratio, diffusion coefficient, hydrogel radius (cylindrical geometry), and time, respectively.

The diffusion coefficient (D) was determined from a plot of S versus $t^{1/2}$, with the slope of the resulting line providing the value of D.

#### 4.4.5. Soil Burial Tests

Dried samples, cut into a disks with a diameter of 9.08 ± 0.15 mm, were first weighed to record their initial mass ($m_{i}$) and then immersed in distilled water for 24 h. The swollen samples were subsequently removed and subjected to soil burial tests at ambient temperature and humidity for a period of 28 days. At the end of this period, the samples were carefully retrieved, washed with distilled water, dried in a laboratory oven at 55 °C until constant mass, and weighed ($m_{f}$) to determine the mass loss. The degree of degradation was calculated as the percentage of mass loss relative to the initial sample mass Equation (18) [79].

(18) $$Mass\;loss\;\left(\%\right)=\frac{m_{i}-m_{f}}{m_{i}}\times 100,$$

#### 4.4.6. Determination of Water Holding Capacity and Water Retention Ratio

To evaluate the effectiveness of the synthesized hydrogel on soil, water holding capacity (WH) and water retention ratio (WR) tests were performed on the same hydrogels before and after NaOH treatment [108,109]. Dry soil was first sifted through a 1 mm mesh sieve. Then, 0.1 g of hydrogel was mixed with 99.9 g of soil, to prepare samples containing 0.1 wt% hydrogel. A control sample of soil without hydrogel was also included. Each 100 g hydrogel/soil mixture was placed in a 300 cm^3^ plastic box covered with a filter paper with a small hole, and the initial mass was recorded. Water was slowly added from the top of the box until the first drop emerged at the bottom. The tests were conducted using both tap water (pH = 7.67) and rainwater (pH = 4.39). For WR study, wet samples prepared for the WH tests were kept at room temperature for 25 days and their mass was recorded daily. WH and WR were calculated using Equations (19) and (20) [107,109]:

(19) $$WH\;\left(\%\right)=\;\frac{m_{2}-m_{1}}{m_{0}}\times 100,$$

(20) $$WR\;\left(\%\right)=\frac{m_{t}-m_{1}}{m_{2}-m_{1}}\times 100$$

where $m_{0}$ is the mass of pure soil, $m_{1}$ is the mass of each dry sample (without water), $m_{2}$ is the mass of each sample immediately after the first water drop appeared at the bottom of the box, and $m_{t}$ is the mass of the sample at specific time intervals (here, recorded daily).

The soil used in this study was characterized by neutral pH and the following composition: organic matter (29–60%), mineral salts (0.5–0.75%), mineral nitrogen (25–50 ppm), water-soluble potassium (52–85 ppm), and soluble phosphorus (32–55 ppm). Although electrical conductivity was not directly measured, based on the low-to-moderate mineral salt content, the soil can be considered non-saline, with an estimated EC within the typical range for agricultural soils (approximately 0.2–1.0 dS/m). These conditions allow for a relevant evaluation of hydrogel performance under realistic agricultural environments.

The pots were re-saturated, and the procedure was repeated for the second wetting-drying cycle.

#### 4.4.7. Structural Investigations by Fourier-Transform Infrared (FTIR) Spectroscopy

The hydrogel structure was characterized by Fourier-transform infrared (FTIR) spectroscopy using a Spectrum 100 instrument (Perkin Elmer, Waltham, MA, USA). Spectra were recorded in attenuated total reflectance (ATR) mode at 4 cm^−1^ resolution over 4000–650 cm^–1^, with 30 scans per sample, and processed and analyzed using Spectrum v. 6.3.2 software.

#### 4.4.8. Morphological Investigations by Scanning Electron Microscopy (SEM)

The morphology of the hydrogels was examined using a FEI Inspect S scanning electron microscope (FEI Co., Ltd., Hillsboro, OR, USA). Hydrogel samples, swollen to equilibrium in distilled water, were freeze-dried before SEM analysis. Cross-sections of the freeze-dried hydrogels were examined after gold coating. SEM imaging was performed at an accelerating voltage of 25 kV with a beam current of 13 pA. A Large Vertical Detector (LVD) was used, and the working distance (WD) between the sample and the objective lens was 34 mm. SEM was used solely for qualitative morphological observations, and no quantitative pore size measurements were performed.

#### 4.4.9. Thermogravimetric and Differential Scanning Calorimetry (TG/DSC) Analysis

Thermograms were recorded on a STA 409 PC Luxx simultaneous TG/DSC system (Netzsch-Gerätebau, Selb, Germany) under nitrogen atmosphere (100 mL/min) using 25 µL aluminum crucibles (≤10 mg). Samples of approximately 2.5 mg were heated from 20 to 590 °C at a constant heating rate of 10 K/min. Calibration and data analysis were performed according to SR EN ISO 11358:2022 [110] and SR EN ISO 11357-1:2023 [111]. Data processing was carried out using Proteus software (version 6.1.0/2019).

#### 4.4.10. Statistical Analysis

All measurements were performed in triplicate. Data were analyzed by one-way ANOVA followed by Tukey’s post hoc test to identify significant differences at *p* < 0.05. Statistical analyses were conducted using Origin Pro 2023.

## Figures and Tables

**Table 1:** First order kinetics: first-order swelling rate constants, $k_{1,s}$ (×10^4^/min^−1^).

PPS	Dose (kGy)	0 M NaOH		0.25 M NaOH		0.5 M NaOH	
		$k_{1,s}$	$R^{2}$	$k_{1,s}$	$R^{2}$	$k_{1,s}$	$R^{2}$
0.1%	10	2.41	0.998	5.21	0.997	6.74	0.988
	15	2.17	0.997	5.51	0.999	6.95	0.990
	20	2.26	0.994	6.18	0.999	9.81	0.997
0.2%	10	2.49	0.997	7.89	0.994	7.06	0.992
	15	1.88	0.997	5.51	0.999	7.40	0.990
	20	2.51	0.997	6.41	0.998	7.05	0.996
0.3%	10	2.15	0.998	5.54	0.994	11.80	0.994
	15	2.17	0.999	5.76	0.998	6.04	0.991
	20	2.73	0.997	6.28	0.997	7.20	0.992

**Table 2:** Second order kinetics: second order swelling rate constants $k_{2,s}$ (×10^8^/g gel (g water min)^−1^), initial swelling rate $r_{0}\;\;($×10^2^/g water (g gel min)^−1^) and equilibrium swelling $S_{eq}$/g water (g gel)^−1^).

PPS	Dose (kGy)	0 M NaOH				0.25 M NaOH				0.5 M NaOH			
		$k_{2,s}$	$r_{0}$	$S_{eq}$	$R^{2}$	$k_{2,s}$	$r_{0}$	$S_{eq}$	$R^{2}$	$k_{2,s}$	$r_{0}$	$S_{eq}$	$R^{2}$
0.1%	10	0.128	20.55	615	0.801	0.291	4.60	865	0.797	0.051	5.50	597	0.864
	15	0.415	33.19	269	0.982	0.794	6.92	427	0.892	0.100	6.06	406	0.889
	20	1.392	50.67	119	0.948	0.983	6.03	411	0.916	0.272	4.75	278	0.978
0.2%	10	0.113	19.66	670	0.716	1.100	3.47	512	0.941	0.111	5.21	416	0.936
	15	0.218	35.98	357	0.944	0.682	6.11	490	0.889	0.137	5.97	350	0.919
	20	1.381	47.63	123	0.985	0.962	5.15	449	0.901	0.166	5.95	318	0.930
0.3%	10	0.076	25.21	722	0.621	0.430	4.95	686	0.837	0.238	3.42	351	0.981
	15	0.250	31.10	359	0.933	0.816	6.18	445	0.887	0.082	6.19	444	0.921
	20	1.395	40.80	133	0.996	0.905	5.38	453	0.903	0.165	6.06	317	0.918

**Table 3:** Swelling parameters: swelling exponents (n) and swelling constants (k).

PPS	Dose (kGy)	0 M NaOH			0.25 M NaOH			0.5 M NaOH		
		n	k	$R^{2}$	n	k	$R^{2}$	n	k	$R^{2}$
0.1%	10	1.04	0.034	0.998	0.97	0.229	0.992	1.01	0.158	0.995
	15	0.89	0.056	0.997	0.90	0.220	0.996	0.94	0.193	0.991
	20	0.75	0.091	0.999	0.92	0.224	0.995	0.88	0.322	0.996
0.2%	10	1.07	0.030	0.997	0.94	0.331	0.993	0.92	0.257	0.990
	15	0.92	0.042	0.997	0.94	0.197	0.995	0.96	0.181	0.991
	20	0.81	0.064	0.998	0.95	0.221	0.992	0.89	0.252	0.990
0.3%	10	1.06	0.024	0.997	1.00	0.185	0.992	0.90	0.393	0.987
	15	0.96	0.038	0.998	0.93	0.207	0.993	0.90	0.241	0.990
	20	0.85	0.056	0.997	0.94	0.226	0.994	0.89	0.255	0.987

**Table 4:** Diffusion parameters: diffusional coefficient (D, ×10^3^ cm^2^·s^−1^).

PPS	Dose (kGy)	0 M NaOH		0.25 M NaOH		0.5 M NaOH	
		D	$R^{2}$	D	$R^{2}$	D	$R^{2}$
0.1%	10	4.30	0.980	15.66	0.969	20.36	0.957
	15	1.10	0.992	5.56	0.985	7.75	0.957
	20	0.33	0.996	7.27	0.979	13.11	0.973
0.2%	10	5.09	0.981	28.11	0.964	13.69	0.957
	15	0.98	0.991	8.45	0.974	10.78	0.963
	20	0.41	0.995	10.11	0.966	7.27	0.971
0.3%	10	2.97	0.981	10.41	0.956	45.18	0.955
	15	1.38	0.987	8.60	0.969	8.12	0.969
	20	0.56	0.996	9.78	0.965	6.90	0.972

## Data Availability

The original contributions presented in this study are included in the article/Supplementary Material. Further inquiries can be directed to the corresponding author.

## Supplementary Materials

The following supporting information can be downloaded at: https://www.mdpi.com/article/10.3390/gels12050395/s1, Figure S1: Swelling of hydrogels prepared with 0.2% PPS: (a) untreated samples; (b) samples treated with 0.25 M NaOH; (c) samples treated with 0.50 M NaOH.; Figure S2: Swelling of hydrogels prepared with 0.3% PPS: (a) untreated samples; (b) samples treated with 0.25 M NaOH; (c) samples treated with 0.50 M NaOH; Figure S3: Water retention ratio (WR) of the hydrogels in tap water for samples irradiated at 10 kGy: (a) control, (b) treated with 0.25 M NaOH, (c) treated with 0.50 M NaOH; and for samples irradiated at 15 kGy: (d) control, (e) treated with 0.25 M NaOH, (f) treated with 0.50 M NaOH; Figure S4: Water retention ratio (WR) of the hydrogels in rainwater for samples irradiated at 10 kGy: (a) control, (b) treated with 0.25 M NaOH, (c) treated with 0.50 M NaOH; and for samples irradiated at 15 kGy: (d) control, (e) treated with 0.25 M NaOH, (f) treated with 0.50 M NaOH.
