# Supplementary material for: Alkaline-Enhanced Poly(Acrylic Acid)/Sodium Alginate/PEO Hydrogels: Structural Modifications and Functional Properties for Agriculture
Source: Gels. 2026 May 2;12(5):395. doi: 10.3390/gels12050395 (PMC13205510; doi:10.3390/gels12050395)
Supplement: Supplementary file 1 [file gels-12-00395-s001.zip › gels-4258597-supplementary.pdf]

Supplementary Materials

2.2. Gel fraction, Network parameters, and Swelling of Hydrogels

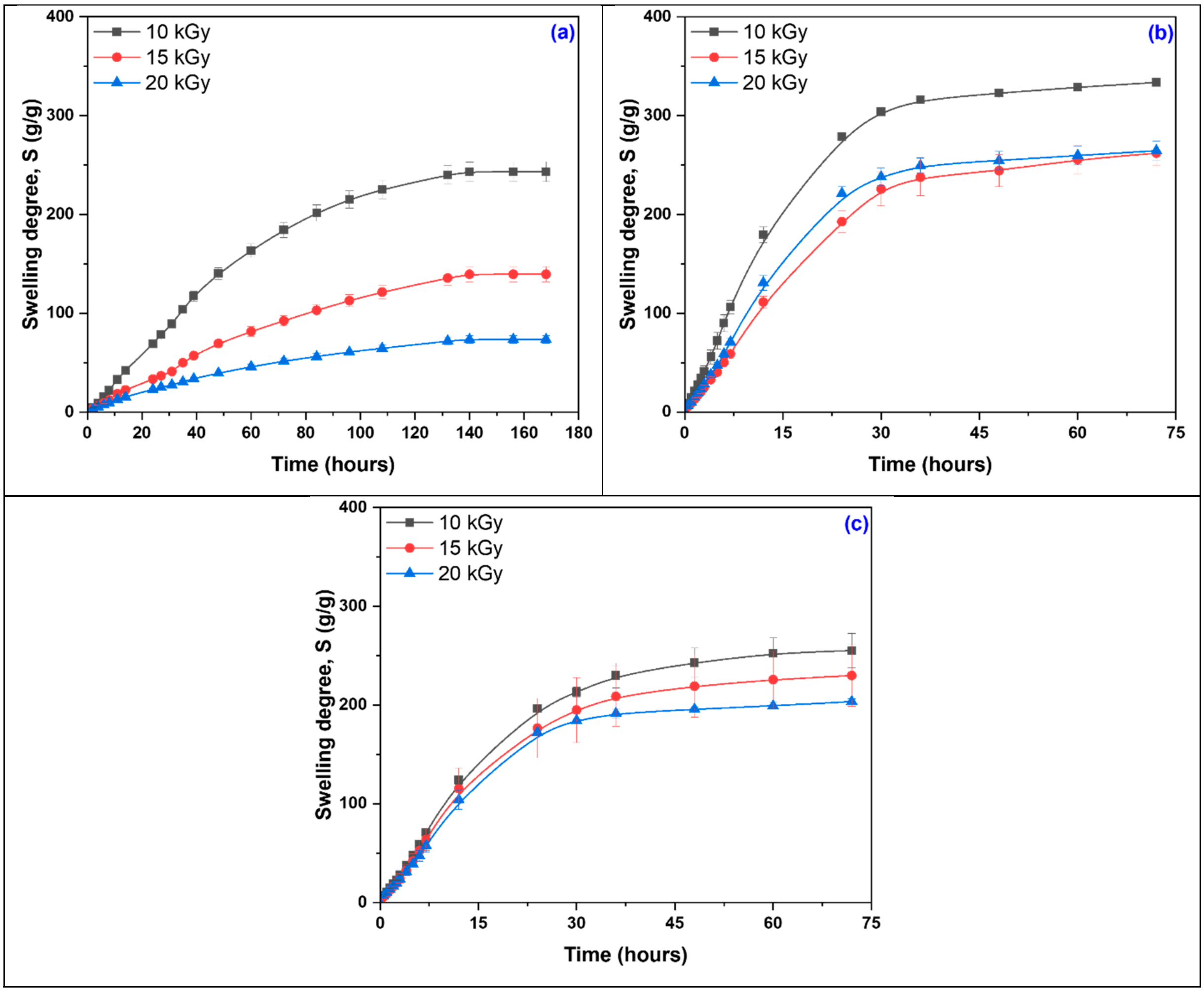

Figure S1. Swelling of hydrogels prepared with 0.2% PPS: **(a)** untreated samples; **(b)** samples treated with 0.25 M NaOH; **(c)** samples treated with 0.50 M NaOH.

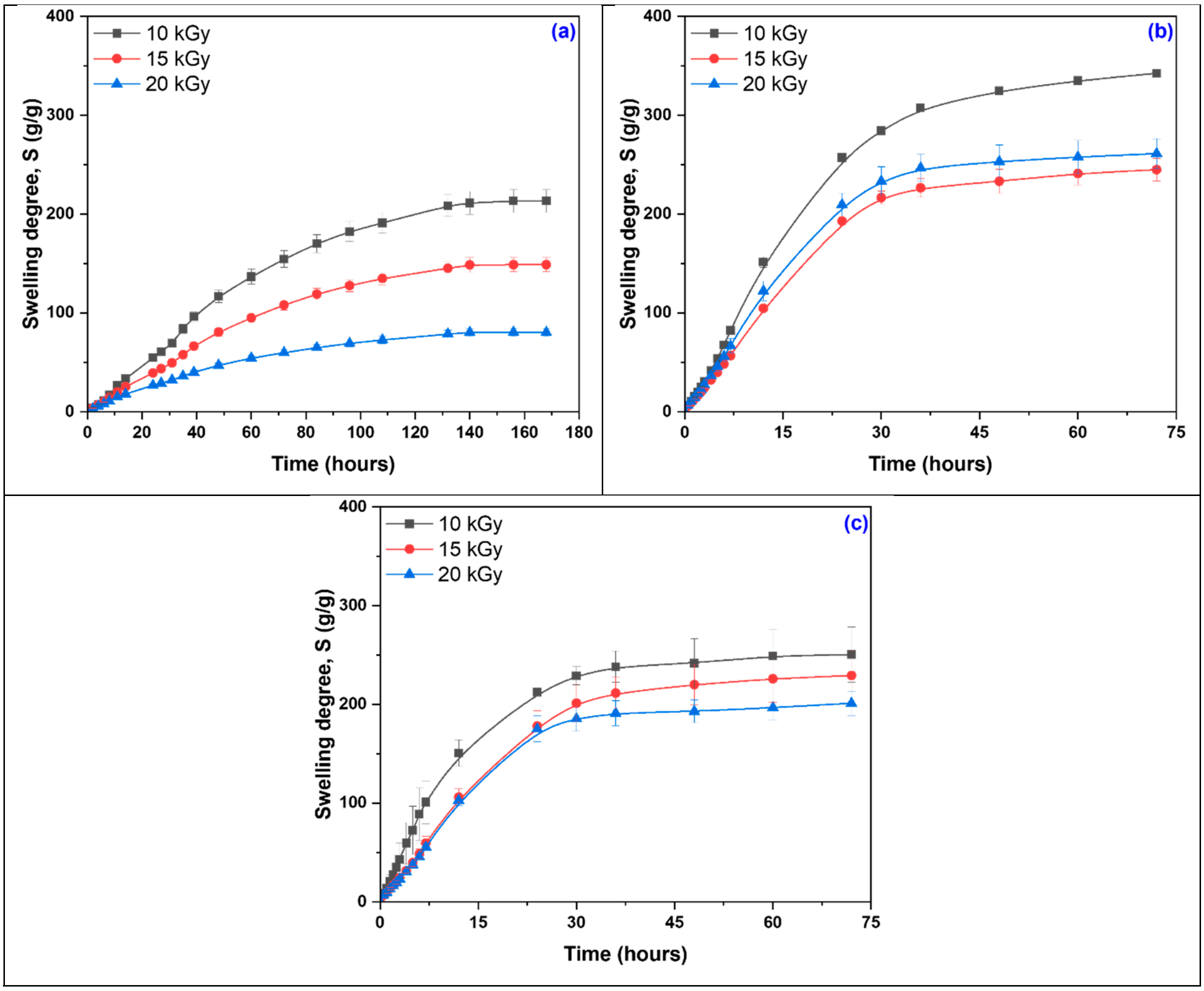

Figure S2. Swelling of hydrogels prepared with 0.3% PPS: **(a)** untreated samples; **(b)** samples treated with 0.25 M NaOH; **(c)** samples treated with 0.50 M NaOH.

2.5. Hydrogel Water-Holding Capacity and Water Retention Ratio

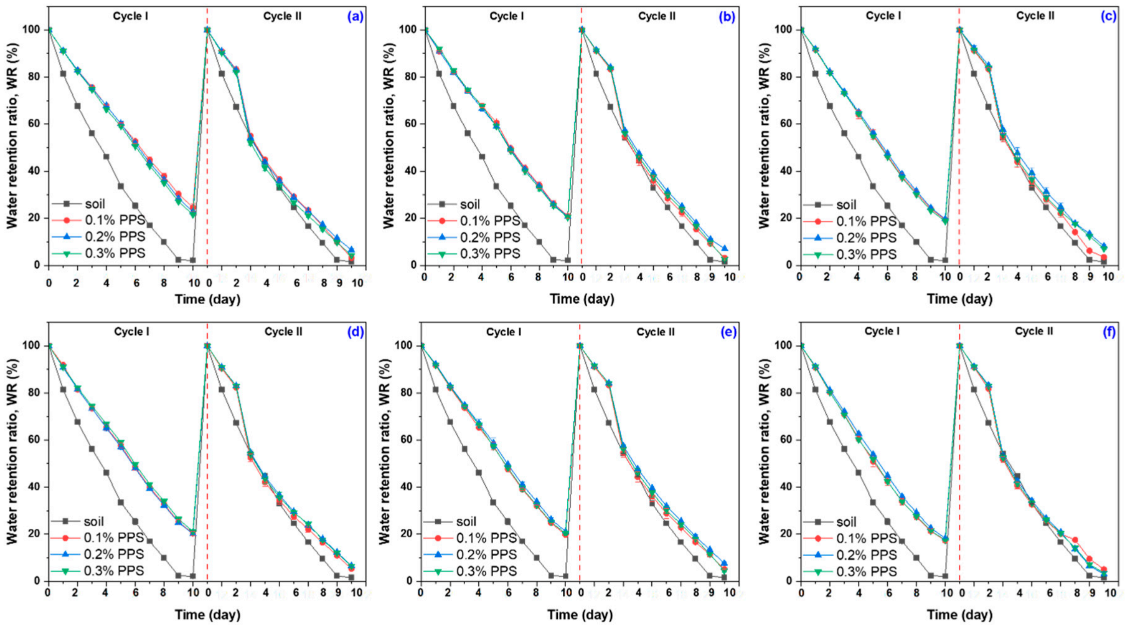

Figure S3. Water retention ratio (WR) of the hydrogels in tap water for samples irradiated at 10 kGy: **(a)** control, **(b)** treated with 0.25 M NaOH, **(c)** treated with 0.50 M NaOH; and for samples irradiated at 15 kGy: **(d)** control, **(e)** treated with 0.25 M NaOH, **(f)** treated with 0.50 M NaOH.

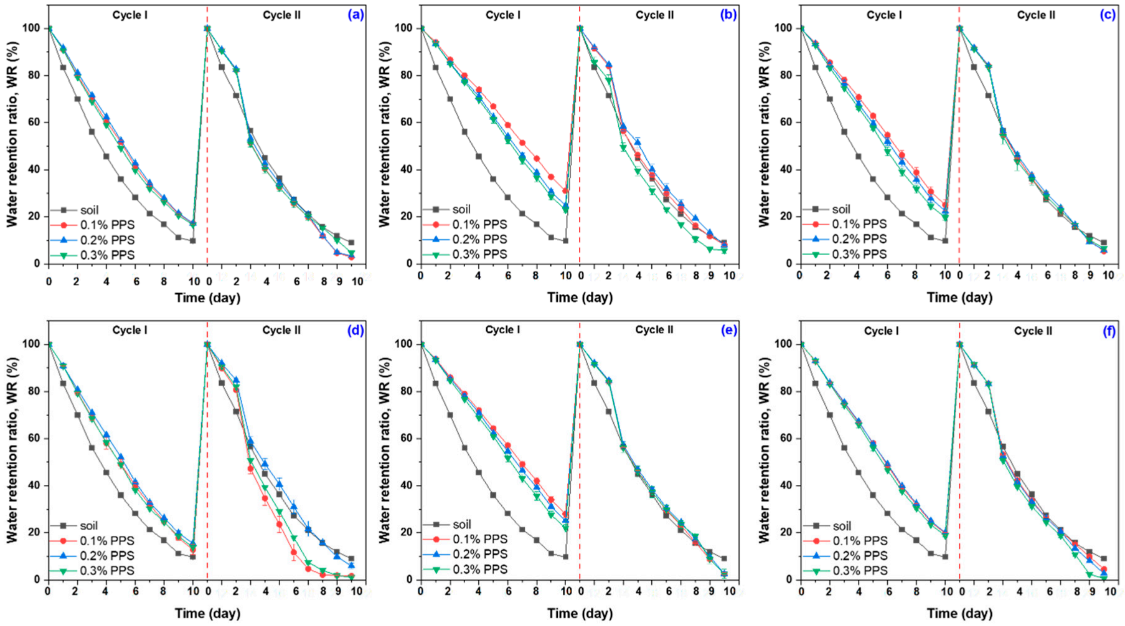

Figure S4. Water retention ratio (WR) of the hydrogels in rainwater for samples irradiated at 10 kGy: **(a)** control, **(b)** treated with 0.25 M NaOH, **(c)** treated with 0.50 M NaOH; and for samples irradiated at 15 kGy: **(d)** control, **(e)** treated with 0.25 M NaOH, **(f)** treated with 0.50 M NaOH.
